# Fisetin Mitigates Ferroptosis and Promotes Remyelination in a Cuprizone Model of Multiple Sclerosis

**DOI:** 10.1007/s11481-025-10260-z

**Published:** 2025-12-09

**Authors:** Nahla E. El-Ashmawy, Naglaa F. Khedr, Nada N. Helmy, Amera O. Ibrahim

**Affiliations:** 1 Department of Biochemistry, Faculty of Pharmacy, Tanta University, El-Geish Street, Medical Campus, Tanta, Gharbia P.O. 31527, Egypt; 2 Department of Pharmacology and Biochemistry, Faculty of Pharmacy, The British University in Egypt, El Sherouk City, Cairo Egypt

**Keywords:** Fisetin, Multiple sclerosis, Cuprizone, NCOA4, Ferroptosis, Neuroprotection

## Abstract

Multiple sclerosis (MS) is a long-lasting autoimmune condition characterized by myelin destruction and neurodegeneration. Research indicates that ferroptosis significantly influences MS pathogenesis, exacerbating neuronal tissue damage. Our study intended to explore the possible neuroprotective role of fisetin (FIS) in cuprizone (CPZ) model of MS and the associated molecular mechanisms. The 9-week experiment comprised a 5-week demyelination period in which C57BL/6 mice were provided with 0.2% w/w CPZ added to rodent chow, followed by a 4-week remyelination period in which mice were fed CPZ-free chow. FIS (80 mg/kg/day) was given by oral gavage to mice daily for 4 weeks starting in the 2nd week of demyelination. For remyelination, FIS was administered daily during the 4 weeks recovery. During demyelination, FIS significantly improved CPZ-induced behavioral and locomotor deficits, as demonstrated by tail suspension test and inverted screen grip strength test. LFB and H & E staining, MBP, GFAP and vimentin immunostaining revealed that FIS treatment significantly improved myelination, alleviated astrogliosis and neuronal injury in CPZ-fed mice throughout both phases. FIS attenuated ferroptosis and neuroinflammation during de- and remyelination as supported by reduced brain iron deposits, IL-1 β, MDA concentrations and restored GPX4. Moreover, FIS significantly downregulated NCOA4 and TfR1 gene expression and TfR1 protein level but upregulated FTH1 gene expression and ferritin protein level. Additionally, FIS upregulated Olig-1 during demyelination, but not remyelination. Fisetin has a potential neuroprotective effect in CPZ model of MS and can be studied as a promising adjuvant therapy to enhance remyelination and mitigate disability in MS patients possibly by modulating ferroptosis pathway.

**Graphical Abstract:**

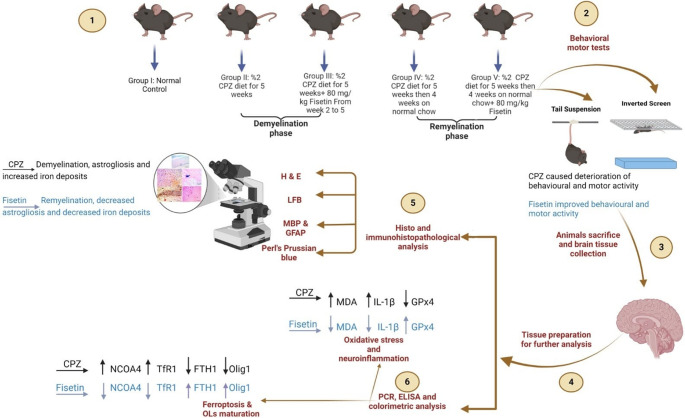

**Supplementary Information:**

The online version contains supplementary material available at 10.1007/s11481-025-10260-z.

## Introduction

Multiple sclerosis (MS) is a neurodegenerative ailment impacting the central nervous system manifested with a chronic course and inflammatory etiology, and it is one of the most common causes of physical disability among young individuals, impacting quality of life, physical capability, and cognitive function (Oh et al. 2018). Globally, the number of individuals affected by MS are estimated at 2.8 million, with a rising rate detected over the past few decades (Portaccio et al. 2024). The disease primarily exhibits a relapsing pattern characterized by irregular episodes of neurological impairment that may initially demonstrate complete improvement. Nevertheless, as the condition progresses, recovery becomes partial, leading to major physical disability (Cree et al. 2021).

Despite introducing multiple anti-inflammatory and/or immuno-modulatory drugs for MS, significant unmet needs persist among most patients with relapsing and progressive disease regarding disease-modifying therapies (DMTs) (Bierhansl et al. 2022). Current research investigates multiple therapeutic targets to address the intricate pathophysiology of MS. The primary aim of current DMTs, especially for relapsing-remitting MS (RRMS) and secondary progressive MS (SPMS), is to alleviate the disease by lessening inflammation, short-term deterioration and the number of relapses but none that substantially influence remyelination (Yang et al. 2022). Recently, over 20 drugs have received FDA approval for the treatment of MS, however, progressive MS targeted treatments remain inadequate, indicating that MS transitions from an initial inflammatory relapsing phase to a later neurodegenerative progressive phase, becoming unresponsive to anti-inflammatory and immunosuppressive therapies (Yang et al. 2022).

The complexity of the pathogenesis of MS makes it challenging to simulate its exact human pathology in animal models and researchers opted to investigate different MS models. Induction of demyelination in several CNS regions can be achieved by ingestion of the copper chelator toxin, cuprizone (CPZ) (Matsushima and Morell 2001). It’s a model widely used to examine therapeutic approaches to either inhibit myelin loss or foster remyelination in MS. Compared to other experimental models of MS with demyelination pathology, the CPZ model offers a distinct advantage in assessing the demyelination and remyelination course, because it shows a prominent similarity to lesions from progressive stages of MS (Münzel and Williams 2013). The deleterious impact of CPZ is linked to the suppression of copper-dependent enzymes in the mitochondria participating in the electron transport chain, resulting in an increase in lipid peroxidation (LPO) products such as acrolein, malondialdehyde (MDA) and 4-hydroxy-2-nonenal (4-HNE) in oligodendrocytes, triggering oligodendrocyte damage, astrogliosis, and toxic demyelination comparable to that studied in MS (Fischer et al. 2013; Signorile et al. 2020). CPZ effect on iron involves disrupting iron homeostasis in the brain leading to increased iron accumulation in brain’s white matter, particularly in regions undergoing demyelination. CPZ interferes with enzymes that require copper as a cofactor, some of which are involved in iron metabolism and antioxidant defense mechanisms (Li et al. 2022).

Dysregulated iron homeostasis, due to the elevated iron content in neurons and oligodendrocytes, is acknowledged as a precipitating factor to neurodegenerative disorders such as MS (David et al. 2022). Ferroptosis, a model of cell death tightly linked to iron, comprises highly reactive labile iron that catalyzes the formation of LPO products by the oxidation of polyunsaturated fatty acids, initiating cellular disruption. Myelin sheaths are made of 30% protein and 70% lipid, which are susceptible to LPO by free radicals, resulting in the formation of MDA and 4-HNE (Barrera et al. 2018). Ferritin is a distinctive protein essential for storing iron within the cell to maintain cellular function and homeostasis and is comprised of 24 subunits of ferritin heavy chain (FTH) and ferritin light chain (FTL) (Kotla et al. 2022). Nuclear receptor coactivator 4 (NCOA4) transfers ferritin to autophagosomes consequently releasing active iron. There is an upregulation in NCOA4 at the peak and progressive stages of experimental MS along with an upregulation in transferrin receptor 1 (TfR1) which enables the uptake of transferrin into cells *via *receptor-mediated endocytosis, subsequently releasing the iron carried on transferrin into the cellular iron pool. These alterations are associated with attenuation of (system xCT, GPX4 and GSH), as well as triggering of lipid peroxidation in MS pathology (Van San et al. 2023).

Plant-based medicine has emerged as a prospective approach for alleviating MS owing to the beneficial pharmacological effects demonstrated in multiple MS animal models (Mohd Sairazi and Sirajudeen 2020; Pourmohammadi et al. 2022; Yuan et al. 2023). Certain plant-based agents have exhibited neuroprotective effects suggesting their prospective therapeutic application in the treatment of neurodegenerative disorders (Mohd Sairazi and Sirajudeen 2020; Rahman et al. 2021).

Fisetin (3,3′,4′,7-tetrahydroxyflavone) is a pharmacologically active plant flavonoid component present mainly in fruits, vegetables and teas, with a notable source in strawberries. Numerous beneficial therapeutic effects of FIS have been documented, encompassing cardioprotective, hepatoprotective, and anti-cancer properties (Liu et al. 2019; Sundarraj et al. 2020; Zhang et al. 2020). Pre-clinical investigations have shown that FIS exhibits antioxidant and neuroprotective effects protecting against trauma-induced brain injury and neurotoxicity elicited by methyl mercury (Jacob and Thangarajan 2018; Zhang et al. 2018). FIS may also be beneficial for alleviating the cognitive deficit and hippocampus synaptic plasticity impairment associated with schizophrenia (Zhan et al. 2021). Recent research proposed that FIS has an impeding effect on ferroptosis through alteration of different pathways (Li et al. 2022; Wang et al. 2023).

Our aim was to investigate the potential neuroprotective role of FIS in CPZ-induced demyelination, highlighting its antioxidant, anti-ferroptotic, and anti-inflammatory effects, along with the molecular mechanisms implicated in the modulation of ferroptosis pathway.

## Materials and methods

### Reagents and Chemicals

Cuprizone (Bis[cyclohexanone]oxaldihydrazone) (purity ≥ 99%; CAS no. 370-81−0) was purchased from (Sigma-Aldrich Co., USA). Sodium carboxymethyl cellulose (Na CMC) was purchased from (Oxford Co., India). Fisetin (purity 90–99%; CAS no. 528-48−3) was purchased from (Naturewill Biotechnology Co., China). Fisetin was suspended in a 0.5% *w/v* Na CMC aqueous solution and thoroughly mixed using a magnetic stirrer until a homogeneous suspension was achieved. All additional chemicals employed in this study were of analytical grade, conforming to the highest commercially available specifications.

### Animals

Forty-eight male C57BL/6 mice, with a weight range of 20–25 g and aged between 6 and 8 weeks were purchased from Medical Experimental Research Center (Mansoura, Egypt). The housing conditions for mice were in wire mesh cages at 23 ± 2 °C temperature and 60 ± 10% humidity experiencing alternate 12 h day/12 h night cycles. The acclimatization period for animals was one week preceding the start of the experiments, and they were allowed unrestricted access to commercial rodent feed and tap water.

All experiments abided by ethical guidelines for animal care and received approval from the Research Ethical Committee of the Faculty of Pharmacy, Tanta University, Egypt (TP/RE/10/24 p-03), as well as the National Institutes of Health guidelines for the care and use of laboratory animals (NIH Publications No. 8023, revised 1978).

### Experimental Design and Animal Groups

**Fig. 1:**
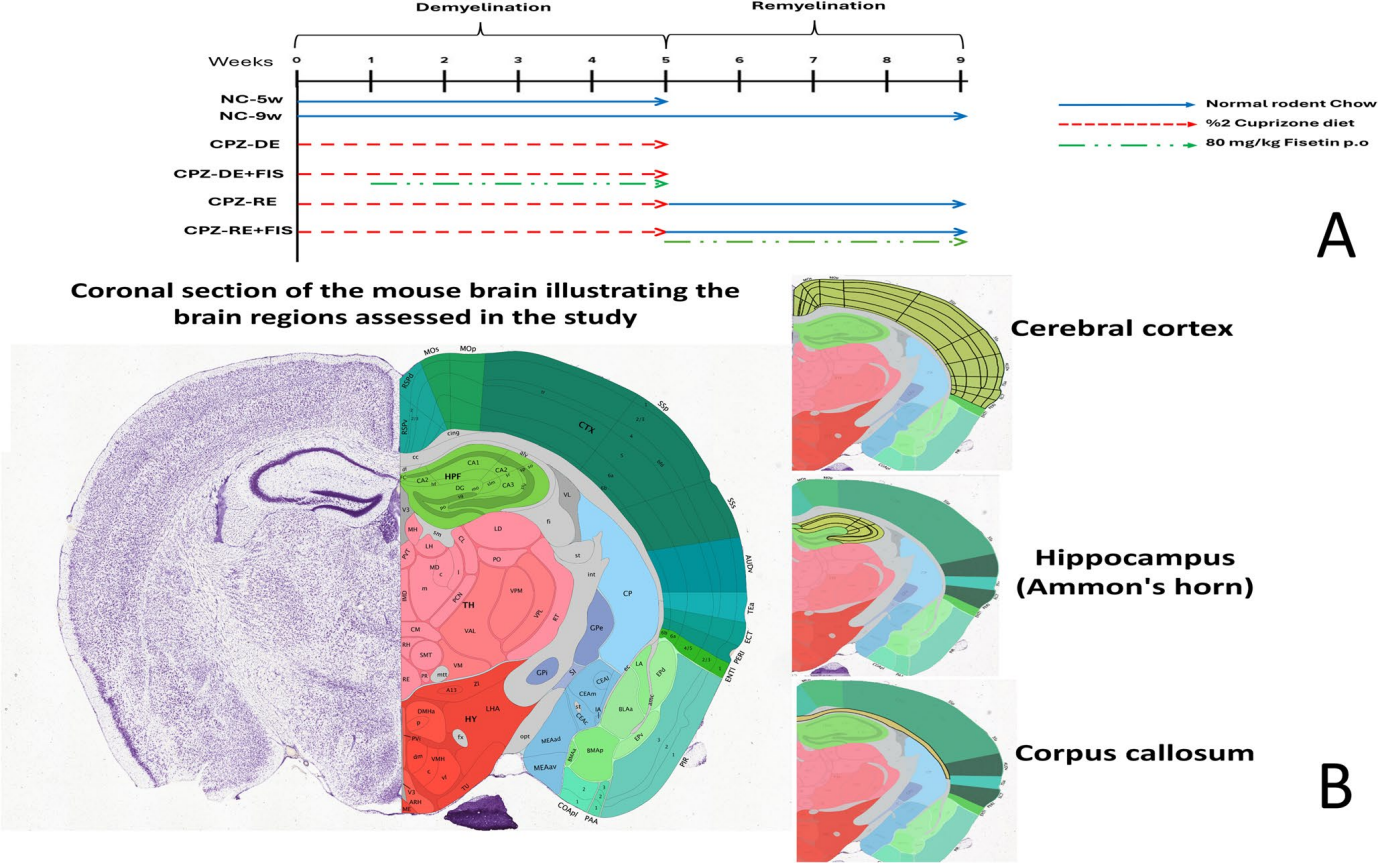
Study design and brain regions assessed in the study 2011 Allen Institute for Brain Science. Allen Mouse Brain Atlas. Available from: https://mouse.brain-map.org

As shown in Figure (1), animals were grouped randomly into one normal control group (*n* = 16) and four equal groups (*n* = 8) receiving different treatments to examine the effect of FIS on both CPZ-induced demyelination and on the remyelination phase after cessation of CPZ as follows:

(i) Normal control (NC) group: Mice were kept on normal rodent chow and given 0.5% *w/v *(Na CMC) by oral gavage as vehicle once daily (10 mL/kg) (Long et al. 2020). At the end of week 5, eight animals were sacrificed to account for NC-5w group and at the end of week 9, the remaining animals were sacrificed to account for NC-9w group.
(ii) Cuprizone-demyelination (CPZ-DE) group: Mice were provided with a diet containing 0.2% *w/w *CPZ blended with normal rodent chow for 5 weeks to induce consistent demyelination in many brain regions (Goldberg et al. 2015).
(iii) Cuprizone-demyelination+ Fisetin (CPZ-DE+FIS) group: Mice were provided with a diet containing 0.2% *w/w* CPZ blended with normal rodent chow for 5 weeks and given FIS by oral gavage at a dose of 80 mg/kg daily starting from the 2^nd^week till the end of week 5 (Yu et al. 2016).
(iv) Cuprizone-remyelination (CPZ-RE) group: Mice were provided with a diet containing 0.2% *w/w *CPZ blended with normal rodent chow for 5 weeks then mice were continued on normal rodent chow for other 4 weeks to allow spontaneous remyelination (week 6 to 9) (Hashem et al. 2022).
(v) Cuprizone-remyelination+ Fisetin (CPZ-RE+FIS) group: Mice were provided with a diet containing 0.2% *w/w* CPZ blended with normal rodent chow for 5 weeks then mice were kept on normal rodent chow and administered FIS by oral gavage at a dose of 80 mg/kg daily for other 4 weeks till the end of week 9.

At the end of week 5 for demyelination groups or week 9 for remyelination groups, animals undergone behavioral tail suspension test and locomotor activity evaluation (inverted screen grip strength test) then all mice were fasted overnight. On the subsequent day, mice were put under anesthesia with sodium pentobarbital (50–90 mg/kg intraperitoneal) (Flecknell 2016) then perfused transcardially using PBS and intact brains were extracted, rinsed with ice-cold saline and the two hemispheres were separated. For biochemical and gene expression analyses, one hemisphere was preserved at −80 °C while the other hemisphere was reserved for histopathological and immunohistochemical evaluations by fixation in 10% neutral buffered formalin.

### Behavioral Assessment and Locomotor Activity

#### Tail Suspension Test (TST)

This test was designed to assess the degree of despair or helplessness. The mice were hung upside down from their tails on a tube positioned approximately 1 cm from the tail tip. The distance from the mouse’s nose to the benchtop measured 10 cm. After thrashing around actively, each animal takes an immobile stance and hangs passively, and the time of immobility after the mice gave up their struggle during the last 4 min of the 5 min observation period was recorded (Tovchiga et al. 2018; Pourmohammadi et al. 2022; Zamali et al. 2024).

#### Inverted Screen Grip Strength Test

The grip strength of mice was assessed using Kondziela’s inverted screen test, employing all four limbs (Zamali et al. 2024; García-Campos et al. 2020; Zhang et al. 2019). The procedure involved positioning the mouse on a (43 cm by 43 cm) wire mesh screen, followed by a 180° rotation of the screen. The mouse was then positioned upside down above a padded cloth and the duration until the mouse fell was recorded in seconds.

### Preparation of Tissue Homogenate

Two brain tissue homogenates were prepared, one in cold buffer (phosphate buffer saline, pH 7.4) in the ratio (1:10 *w/v*) for the assay of GPX4, IL-1β, TfR1 and ferritin and another in (50 mM potassium phosphate, pH 7.5) in the ratio (1:10 *w/v*) for the assay of MDA, using tissue homogenizer (WiseTis homogenizer HG-15D Witeg Labortechnik GmbH, Germany). Then the homogenates were centrifuged at 3000 r.p.m (Sigma centrifuge 3K15 SIGMA Laborzentrifugen GmbH, Germany) for 20 min at 4 C˚ and the supernatant was separated for further analyses.

### Determination of Brain IL-1β, GPX4 (EC 1.11. 1.12), TfR1 and Ferritin by ELISA

GPX4 and IL-1β protein levels were assayed using ELISA kits (Shanghai Sunred Biological Technology Co., China) with a sensitivity range 0.08–15 ng/mL, Cat. No. 201-02−1677 for GPX4, and with a sensitivity range 0.08–15 pg/mL, Cat. No. 201–020193 for IL-1β, according to the manufacturer’s protocol. TfR1 and ferritin protein levels were assayed using ELISA kits (Elabscience Co., USA) with a sensitivity range 0.63–40 ng/mL, Cat. No. E-EL-M1186 for TfR1, and with a sensitivity range 1.56–100 ng/mL, Cat. No. E-EL-M0491 for ferritin, according to the manufacturer’s protocol.

### Determination of MDA Level in Mice Brain

Lipid peroxidation levels were assessed colorimetrically as thiobarbituric acid reactive substances (TBARS) using a kit obtained from Biodiagnostic, Egypt, following the kit’s booklet instructions. Measured values were expressed as nmol MDA/g tissue (Ohkawa et al. 1979).

### Relative Gene Expression of NCOA4, FTH1, TfR1 and Olig-1 by real-time PCR

Invitrogen™ TRIzol Reagent (Cat. No. 15596026, Life Technologies., USA) was used to extract Total RNA from brain tissue homogenates. The purity of obtained RNA was validated spectrophotometrically at 260/280 nm by nanodrop (Wilfinger et al. 2006). 1 µg total RNA was reverse transcribed to cDNA using QuantiTect Reverse Transcription Kit (Cat. No. 205311, Qiagen., USA). 30 ng of the cDNA was used for quantitative PCR utilizing Maxima SYBR Green/Fluorescein qPCR Master Mix (Thermo Fisher Scientific Baltics UAB, Lithuania) (Cat. No. K0241) as clarified by the instruction booklet. Primer sequences (Biosearch Technologies Co., USA) were as follows:

**Table:**
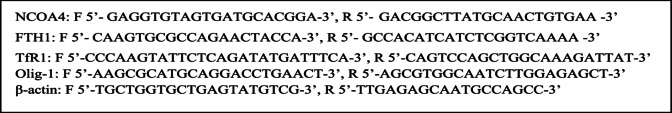

F: Forward primer, R: Reverse primer.

The PCR procedure was an initial polymerase activation at 95 °C for 10 min, then 40 cycles of denaturation at 95 °C for 5 s, annealing at 60 °C for 30 s, and extension at 72 °C for 30 s. The reaction mixture was then placed in a qRT-PCR equipment, Rotor-Gene Q (Qiagen, USA). All samples were assessed and normalized to the housekeeping gene (β-actin) level. Threshold cycle (Ct) values were attained, and transcript levels were quantified and described as relative quantification (RQ) or the relative expression of target genes utilizing the 2^−ΔΔCT^formula as previously explained by (Vandesompele et al. 2009). BLAST was used to compare the primer and template sequences to sequences databases to check if they were unique https://blast.ncbi.nlm.nih.gov/Blast.cgi.

### Histopathological Examination

Fixation of brain tissue samples was applied using 10% neutral buffered formalin. Afterwards, they were embedded into paraffin blocks. A sliding microtome (Leica RM2155, Germany) was used to slice 5-µm thick coronal brain sections from the paraffin blocks. Afterwards, some tissue sections were deparaffinized and stained with hematoxylin and eosin (H&E) stain, whereas other sections were processed for LFB staining, or Prussian blue staining as follows:

#### Luxol Fast Blue (LFB) Staining

Luxol fast blue (LFB) was used for the analysis of demyelination in corpus callosum (CC), hippocampus (HC) and cerebral cortex (CTX) regions. The tissue sections underwent rehydration in 95% ethanol before submersion in a 0.01% LFB solution all night at 55 °C then differentiation of tissue sections was performed using a 0.05% lithium carbonate and 70% ethyl alcohol solution. Samples fixation and processing protocols were employed according to (Culling 2013). The myelinated area was detected under an Olympus light microscope (Olympus, Tokyo, Japan), photographed, and quantified as percentage using ImageJ analysis software at 400x magnification by an expert pathologist who is blinded to the experiment.

#### Prussian Blue (PB) Staining

Prussian blue staining was carried out to measure non-heme iron deposition. Briefly, paraffin slices were immersed in Perl’s staining solution for 30 min. Then, rinsed with distilled water and counterstained with nuclear fast red solution for 3 min, so the nuclei appeared red, and the cytoplasm appeared pink while the iron labeled cells appeared blue (Meguro et al. 2007). The PB deposition was detected under a light microscope (Olympus, Tokyo, Japan). The percentage of positive areas for PB staining was assessed using ImageJ analysis software at 400x magnification by an expert pathologist who is blinded to the experiment.

### Immunohistochemistry for GFAP, MBP and Vimentin

Brain tissue sections were de-waxed and rehydrated by gradual descending grades of alcohol, then washed twice in PBS. At room temperature, the sections were immersed for 10 min in hydrogen peroxide (3%) to inactivate endogenous peroxidase. Then sections were washed with PBS and underwent incubation with anti-glial fibrillary acidic protein (GFAP) rabbit monoclonal antibody (1:100, Cat. No. DL98555A) or with anti-myelin basic protein (MBP) rabbit monoclonal antibody (1:200, Cat. No. DL96936A) or with vimentin rabbit polyclonal antibody (1:200 Cat. No. DL95312A) from Wuxi Donglin Sci &Tech Development Co., China, all night at 4 °C, then underwent incubation with biotinylated goat anti rabbit antibody for 15 min. Lastly, sections underwent incubation with diaminobenzidine (DAB) for 10 min then counterstained with hematoxylin and cover slipped to commence microscopic analysis. Immunostaining was assessed using ImageJ analysis software at 400x magnification by an expert pathologist who is blinded to the experiment.

### Statistical Analysis

All statistical analyses were conducted applying GraphPad Prism 8.0 software (GraphPad Software, USA). The Shapiro–Wilk test was employed to evaluate the normality of data distribution. The data are expressed as mean ± standard deviation (SD). One-way analysis of variance (ANOVA) and two-way ANOVA were used for statistical intergroup comparisons, employing Fisher’s least-significant differences (LSD) method for pairwise group comparisons. The threshold for statistical significance was defined at *p* < 0.05.

## Results

### Effect of Fisetin on Behavioral and Locomotor Abnormalities

Table 1 demonstrates that CPZ-fed animals developed behavioral and locomotor defects, shown by tail suspension and inverted screen grip strength tests. The CPZ-DE group displayed a significant (*p* < 0.0001) rise in immobility time by 140% vs. the NC-5w group. CPZ-intoxication significantly (*p* < 0.0001) decreased time to hold the inverted screen (latency to fall) in grip strength test by 77% compared to NC-5w group. Fisetin treatment in CPZ-DE + FIS group significantly (*p* < 0.0001) decreased immobility time by 46% and significantly (*p* = 0.0004) improved time to hold the inverted screen by 153% vs. the CPZ-DE group. However, this improvement remained significantly different from NC-5w mice.

**Table 1:** Effect of Fisetin on behavioral and locomotor abnormalities

			Demyelination phase			Remyelination phase		
**Test**	**NC-5w**	**CPZ-DE**		**CPZ-DE + FIS**	**NC-9w**		**CPZ-RE**	CPZ-RE + FIS
TST immobility time (sec)	58.84 ± 7.25	141.4 ± 10.11^a^		76.45 ± 10.63^a, b^	62.38 ± 4.96		96.56 ± 8.27^c^	66.42 ± 8.43^d^
Latency to fall (sec)	69.27 ± 17.11	15.95 ± 7.88^a^		40.48 ± 14.58^a, b^	72.67 ± 15.70		29.77 ± 10.43^c^	41.20 ± 9.68^c^

Immobility time in the last 4 min of the 5 min of TST (sec) and latency to fall in inverted screen test for grip strength (sec).

Data is represented as mean ± SD (*n* = 8). Significance was set at *p* < 0.05, *a* significant vs. NC-5w group, *b* significant vs. CPZ-DE group, *c* significant vs. NC-9w group, *d* significant vs. CPZ-RE group

*NC-5w* 5 week-normal control group, *NC-9w* 9 week-normal control group, *CPZ-DE* Cuprizone-demyelination group, *CPZ-DE + FIS* Cuprizone-demyelination + Fisetin group, *CPZ-RE* Cuprizone-remyelination group, *CPZ-RE + FIS* Cuprizone-remyelination + Fisetin group, *TST* Tail suspension test

For remyelination (4 weeks after CPZ cessation), CPZ-RE + FIS group showed significant (*p* < 0.0001) reduction in immobility time by 31% compared to CPZ-RE group and showed non-significant difference from NC-9w group, while showing non-significant difference in grip strength test from CPZ-RE group (Table 1).

### Effect of Fisetin on Inflammation and Oxidative Stress in Mice Brain

Table 2 displays that during demyelination, CPZ-intoxication significantly (*p* < 0.0001) increased MDA and IL-1β concentration by 5-fold and 3-fold, while significantly (*p* < 0.0001) diminishing GPX4 concentration by 73% vs. NC-5w group. FIS administration in CPZ-DE + FIS group significantly (*p* < 0.0001) reduced MDA and IL-1β concentration by 65% and 52%, while significantly (*p* < 0.0001) increasing GPX4 concentration by 141% vs. CPZ-DE group.

**Table 2:** Effect of Fisetin on inflammation (IL-1β), oxidative stress (GPX4 & MDA) and ferroptosis (TfR1, ferritin)

			Demyelination phase			Remyelination phase		
**Parameters**	**NC-5w**	**CPZ-DE**		**CPZ-DE + FIS**	**NC-9w**		**CPZ-RE**	CPZ-RE + FIS
GPX4 ng/g tissue	11.63 ± 0.86	3.08 ± 1.09^a^		9.83 ± 0.7^a, b^	10.94 ± 1.29		7.45 ± 0.85^c^	9.897 ± 1.06^c, d^
MDA nmol/g tissue	27.14 ± 12.38	146.4 ± 22.15^a^		50.7 ± 9.17^a, b^	34.85 ± 9.46		102.8 ± 11.29^c^	54.17 ± 4.52^c, d^
IL-1β pg/g tissue	65.6 ± 5.28	191.9 ± 30.23^a^		91.52 ± 7.16^a, b^	68.61 ± 2.94		130.6 ± 6.51^c^	76.49 ± 5.42^d^
TfR1 ng/g tissue	21.60 ± 8.93	138.2 ± 15.74^a^		65.53 ± 13.64^a, b^	24.58 ± 7.77		86.14 ± 11.62^c^	59.62 ± 8.31^c, d^
Ferritin ng/g tissue	413.1 ± 70.52	121.7 ± 36.96^a^		261.8 ± 36.8^a, b^	359.7 ± 44.93		171.8 ± 37.94^c^	273.9 ± 56.31^c, d^

Data is represented as mean ± SD (*n* = 8). Significance at *p* < 0.05; a: significant vs. NC-5w group, b: significant vs. CPZ-DE group, c: significant vs. NC-9w group, d: significant vs. CPZ-RE group.

*NC-5w* 5 week-normal control group, *NC-9w* 9 week-normal control group, *CPZ-DE* Cuprizone demyelination group, *CPZ-DE + FIS* Cuprizone-demyelination + Fisetin group, *CPZ-RE* Cuprizone-remyelination group, *CPZ-RE + FIS* Cuprizone-remyelination + Fisetin group, *GPX4* Glutathione peroxidase 4, *MDA* Malondialdehyde, *IL-1β* Interleukin 1- beta. *TfR1* Transferrin receptor 1

For remyelination groups, FIS significantly (*p* < 0.0001) reduced MDA and IL-1β concentration by 47% and 41% in CPZ-RE + FIS group, while significantly (*p* < 0.0001) elevating GPX4 concentration by 32% vs. CPZ-RE group. CPZ-RE + FIS group showed a significant difference from NC-9w group in MDA and GPX4 concentrations, but a non-significant difference in IL-1β concentration (Table 2).

### Effect of Fisetin on Ferroptosis Related Biomarkers in Mice Brain

Table 2 presents that during demyelination, CPZ-intoxication significantly (*p* < 0.0001) increased TfR1 concentration by 6.6-fold, while significantly (*p* < 0.0001) reducing ferritin concentration by 70% vs. NC-5w group. FIS treatment in CPZ-DE + FIS group significantly (*p* < 0.0001) reduced TfR1 concentration by 53%, while significantly (*p* < 0.0001) augmenting ferritin concentration by 115% vs. CPZ-DE group.

For remyelination groups, FIS significantly (*p* < 0.0001) lowered TfR1 concentration by 31.4% in CPZ-RE + FIS group, while significantly (*p* = 0.0001) raising ferritin concentration by 60% vs. CPZ-RE group. CPZ-RE + FIS group showed a significant difference vs. NC-9w group in both TfR1 and ferritin levels (Table 2).

**Fig. 2:**
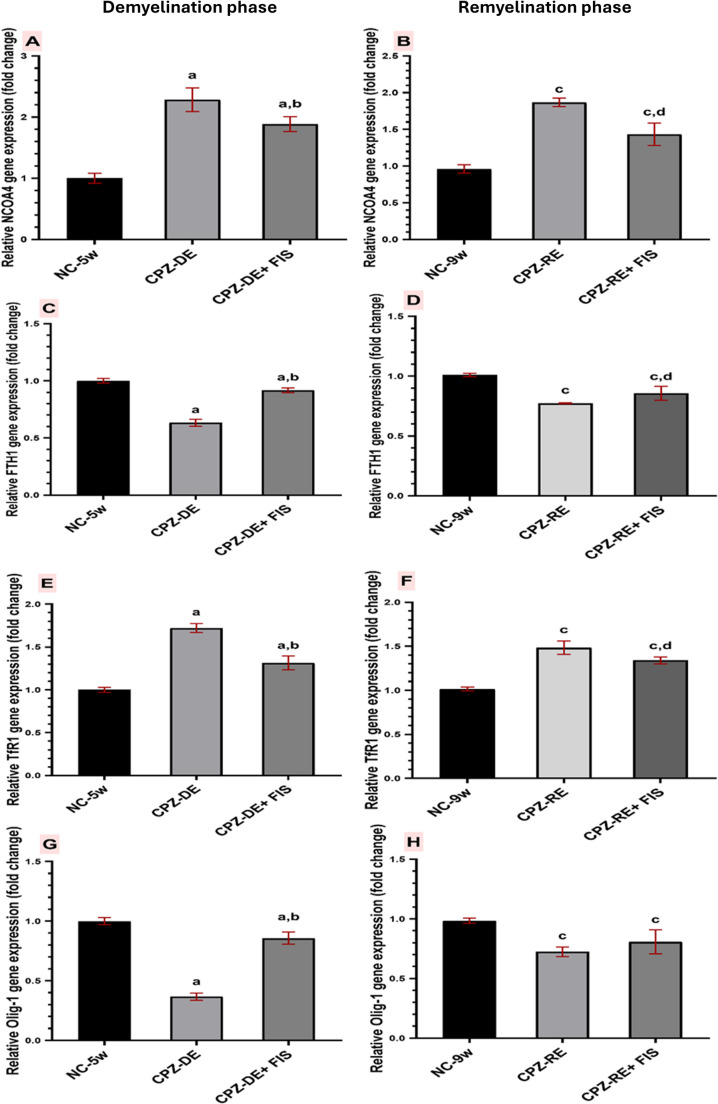
Effect of fisetin on relative mRNA expression of ferroptosis related biomarkers and oligodendrocytes maturation. (**A**) NCOA4 mRNA expression. (**B**) FTH1 mRNA expression. (**C**) Tfr1 mRNA expression. (**D**) Olig-1 mRNA expression. All expressed as relative mRNA expression of n-fold difference to reference gene β-actin. Data shown as mean ± SD (*n* = 8), Significance at *p* < 0.05; **a**: significant vs. NC-5w group, **b**: significant vs. CPZ-DE group, **c**: significant vs. NC-9w group, **d**: significant vs. CPZ-RE group. NC-5w: 5 week-normal control group; NC-9w: 9 week-normal control group; CPZ-DE: Cuprizone demyelination group; CPZ-DE + FIS: Cuprizone-demyelination + Fisetin group; CPZ-RE: Cuprizone-remyelination group; CPZ-RE + FIS: Cuprizone-remyelination + Fisetin group; NCOA4: Nuclear receptor coactivator 4; TfR1: Transferrin receptor 1; FTH1: Ferritin heavy chain 1; Olig-1: Oligodendrocyte transcription factor 1

Figure 2A and E show significant (*p* < 0.0001) 2.3 folds up-regulation in NCOA4 gene expression and significant (*p* < 0.0001) 1.7 folds up-regulation in TfR1 gene expression in CPZ-DE group vs. NC-5w group. Fisetin treatment significantly (*p* = 0.0039) downregulated NCOA4 gene expression to 82% and significantly (*p* < 0.0001) downregulated TfR1 gene expression to 75% vs. CPZ-DE group.

For remyelination groups (Fig. 2B and F), significant (*p* = 0.0002) 1.8 folds up-regulation in NCOA4 gene expression and significant (*p* = 0.0002) 1.5 folds up-regulation in TfR1 gene expression occurred in CPZ-RE group vs. NC-9w group. CPZ-RE + FIS group showed significant (*p* = 0.0022) downregulation in NCOA4 gene expression to 76% of CPZ-RE group, and significant downregulation (*p* = 0.0145) in TfR1 gene expression to 90% of CPZ-RE group.

As shown in Fig. 2C, the CPZ-DE group showed significant (*p* < 0.0001) down-regulation in FTH1 gene expression to 63% of the NC-5w group. Fisetin administration significantly (*p* < 0.0001) up-regulated FTH1 gene expression by 1.4 folds vs. the CPZ-DE group. For remyelination groups, CPZ-RE group showed significant (*p* = 0.001) down-regulation in FTH1 gene expression to 77% of the NC-9w group, while CPZ-RE + FIS group significantly (*p* = 0.0103) upregulated FTH1 expression by 1.1 folds vs. the CPZ-RE group (Fig. 2D).

### Effect of Fisetin on Oligodendrocytes Maturation (Olig-1) in Mice Brain

Figure 2G revealed that CPZ-feeding significantly (*p* < 0.0001) down-regulated Olig-1 gene expression to 36% of the NC-5w group. FIS administration significantly (*p* < 0.0001) up-regulated Olig-1 gene expression by 2.3 folds compared to CPZ-DE group. For remyelination groups, CPZ-RE group showed significant (*p* = 0.009) down-regulation in Olig-1 gene expression to 72% of the NC-9 group. CPZ-RE + FIS group up-regulated Olig-1 gene expression vs. CPZ-RE group but it wasn’t statistically significant (Fig. 2H).

### Histological Findings in Mice Brain in Cerebral cortex, Hippocampus and Corpus Callosum of Mice Brain

**Fig. 3:**
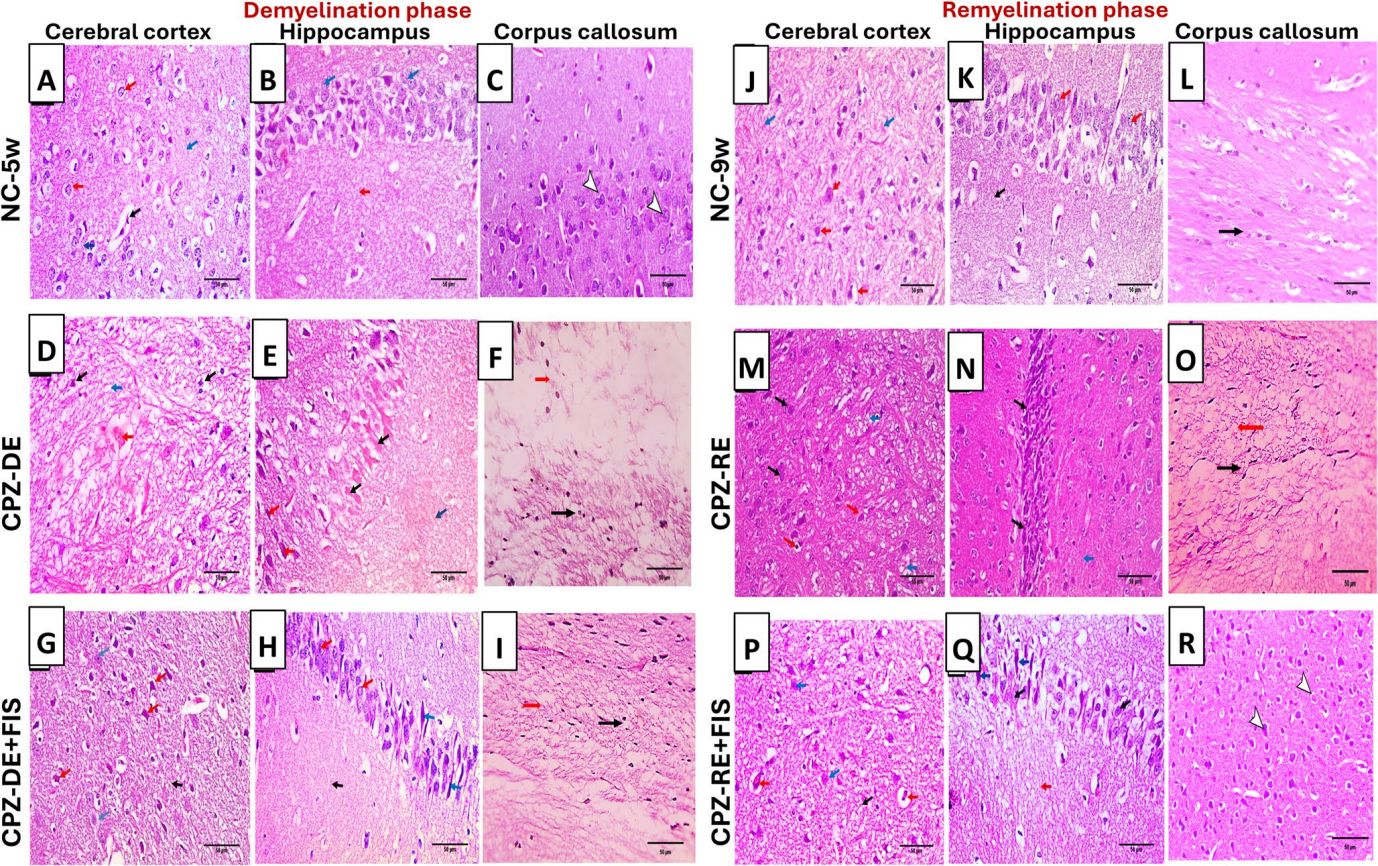
Representative photomicrographs of cerebral cortex, hippocampus and corpus callosum (H&E) 400x magnification, scale bar 50 μm.(**A**) CTX in NC-5w showing normal oligodendroglial cells with fried egg appearance [surrounded by clear halo] (red arrows) and normal blood vessel (black arrow) with glial stroma (blue arrow). (**B**) HC in NC-5w showing normal pyramidal layer of packed rounded neurons with vesicular nuclei (blue arrows) and glial stroma (red arrow). (**C**) CC in NC-5w showing densely packed, and parallel arranged fibers with normal neuronal cells (arrowhead) (**D**) CTX in CPZ-DE showing necrosis (red arrow), pyknotic oligodendroglial cells (black arrows) with highly edematous stroma (blue arrow). (**E**) HC in CPZ-DE showing necrotic neurons (black arrows) and neurons with dense pyknotic nuclei (red arrows) in degenerated edematous stroma (blue arrow). (**F**) CC in CPZ-DE showing degenerated nerve fibers (red arrow) with pyknotic nuclei (black arrow). (**G**) CTX in CPZ-DE + FIS showing pyknotic oligodendroglial cells (red arrows), normal cells (blue arrows) with fibrillary glial stroma (black arrow). (**H**) HC in CPZ-DE + FIS showing packed neurons with vesicular nuclei (red arrows) and degenerated neurons with pyknotic nuclei (blue arrows) in mild edematous stroma (black arrow). (**I**) CC in CPZ-DE + FIS showing packed neurons with less degenerated nerve fibers (black arrow) and pyknotic nuclei (red arrows). (**J**) CTX in NC-9w showing normal oligodendroglial cell (red arrows) with glial fibrillary stroma (blue arrows). (**K**) HC in NC-9w showing normal pyramidal layer of packed rounded neurons with vesicular nuclei (red arrows) and glial stroma (black arrow). (**L**) CC in NC-9w showing oligodendrocytes nuclei (black arrow) that appear well defined, rounded, darkly stained and arranged in longitudinal rows parallel to the nerve fibers (**M**) CTX in CPZ-RE showing pyknotic oligodendroglial cells (red arrows) and normal cells (black arrows) in moderate edematous stroma. (**N**) HC in CPZ-RE showing packed neurons with pyknotic nuclei (black arrows) in mild edematous stroma (blue arrow). (**O**) CC in CPZ-RE showing packed neurons with less degenerated nerve fibers (black arrow) and pyknotic nuclei (red arrows). (**P**) CTX in CPZ-RE + FIS showing normal oligodendroglial cells (blue arrows) and blood vessels (red arrows) in mild edematous stroma (black arrow). (**Q**) HC in CPZ-RE + FIS showing packed neurons with vesicular nuclei (blue arrows) and degenerated neurons with pyknotic nuclei (black arrows) in mild edematous stroma (red arrow). (**R**) CC in CPZ-RE + FIS in showing marked decrease in neuronal vacuolation and well defined, rounded, darkly stained neuronal cells (arrowheads). NC-5w: 5 week-normal control group; NC-9w: 9 week-normal control group; CPZ-DE: Cuprizone-demyelination group; CPZ-DE + FIS: Cuprizone-demyelination + Fisetin group; CPZ-RE: Cuprizone-remyelination group; CPZ-RE + FIS: Cuprizone-remyelination + Fisetin group

Figure 3 shows H&E-stained sections of cerebral cortex (CTX) and hippocampus (HC). The CTX in NC-5w group showed normal oligodendroglial cells with fried egg appearance and normal sized blood vessels surrounded by glial stroma. The HC showed a normal pyramidal layer of tightly packed rounded neurons with large vesicular nuclei surrounded by glial stroma and The CC showed densely packed, and parallel arranged fibers with normal neuronal cells (Figs. 3A, B & C). The CTX in CPZ-DE group showed necrosis, pyknotic oligodendroglial cells with highly edematous stroma, the HC section showed necrotic neurons with dense pyknotic nuclei surrounded by degenerated edematous stroma and the CC section showed degenerated nerve fibers with pyknotic nuclei (Fig. 3D, E & F). FIS treatment in CPZ-DE + FIS group showed improvement with fewer pyknotic oligodendroglial cells and normal cells surrounded by fibrillary glial stroma in CTX, packed neurons with vesicular nuclei and fewer degenerated neurons with pyknotic nuclei and mild edematous stroma in HC and packed neurons with less degenerated nerve fibers and pyknotic nuclei in CC (Fig. 3G, H & I).

For remyelination groups, CTX in NC-9w group showed normal oligodendroglial cells surrounded by glial fibrillary stroma, HC showed a normal pyramidal layer of packed rounded neurons with large vesicular nuclei in glial stroma and CC exhibited oligodendrocytes nuclei that appear well defined, rounded, darkly stained and arranged in longitudinal rows parallel to the nerve fibers. (Fig. 3J, K & L). CPZ-RE group showed improvement vs. CPZ-DE group with fewer pyknotic oligodendroglial cells and more normal cells in moderate edematous stroma in CTX, packed neurons with pyknotic nuclei in mild edematous stroma in HC and packed neurons with less degenerated nerve fibers and less pyknotic nuclei in CC (Fig. 3M, N & O). CPZ-RE + FIS group showed normal oligodendroglial cells and blood vessels in mild edematous stroma in CTX, packed neurons with vesicular nuclei and fewer degenerated neurons with pyknotic nuclei in mild edematous stroma in HC and marked decrease in neuronal vacuolation with well defined, rounded, darkly stained neuronal cells in CC (Fig. 3P, Q & R).Supplementary Figure 2 (Representative photomicrographs of CTX, HC and CC (H&E) in 400x and 200x magnifications) is linked in the supplementary material.

### Effect of Fisetin on Brain Myelination State

**Fig. 4:**
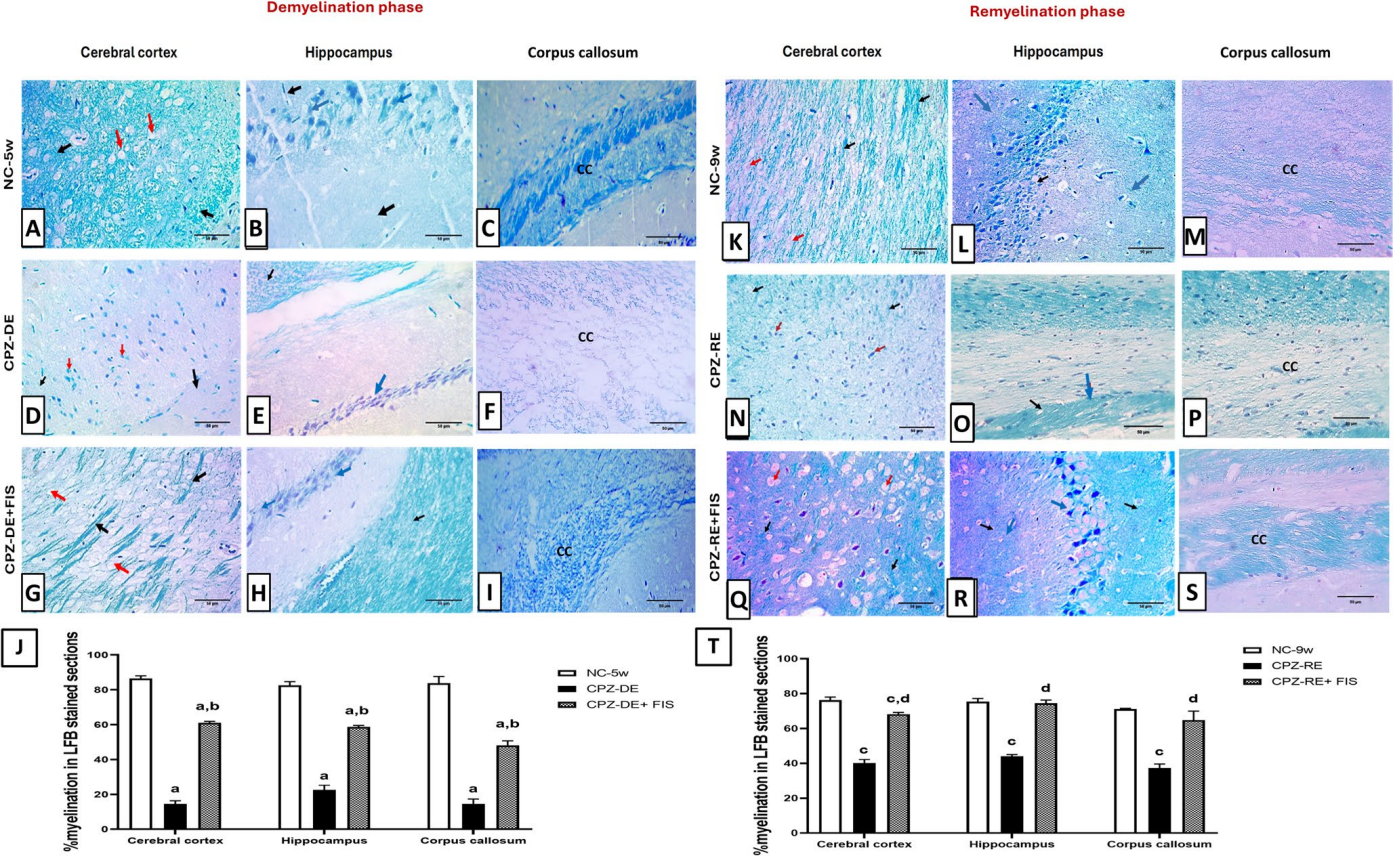
Effect of fisetin on brain myelination state by LFB staining. (**A**-**I**, **K**-**S**): Photomicrographs of brain sections showing cerebral cortex, hippocampus and corpus callosum of studied groups stained with luxol fast blue (LFB) 400x magnification, scale bar 50 μm. (**J**,** T**): Statistical analysis of % myelination in demyelination and remyelination stages. For CTX, red arrow (oligodendroglial cells) and black arrow (nerve fibers). For HC, blue arrow (neurons) and black arrow (nerve fibers). Data shown as mean ± SD (*n* = 3). Significance at *p* < 0.05; a: significant vs. NC-5w group, b: significant vs. CPZ-DE group, c: significant vs. NC-9w group, d: significant vs. CPZ-RE group. NC-5w: 5 week-normal control group; NC-9w: 9 week-normal control group; CPZ-DE: Cuprizone-demyelination group; CPZ-DE + FIS: Cuprizone-demyelination + Fisetin group; CPZ-RE: Cuprizone-remyelination group; CPZ-RE + FIS: Cuprizone-remyelination + Fisetin group

Histochemical staining with LFB revealed that CPZ-fed mice showed a significant (*p* < 0.0001) reduction in myelinated areas in CTX surrounding disorganized oligodendroglial cells by 83% and nerve fibers in HC by 72% and CC by 82%, compared to NC-5w mice (Fig. 4). Treatment with FIS significantly (*p* < 0.0001) improved myelinated areas in nerve fibers surrounding packed oligodendroglial cells in CTX by 4-folds, in HC by 3-folds and in CC by 3.3- folds compared to CPZ-DE group.

For remyelination groups, CPZ-RE + FIS group showed a significant (*p* < 0.0001) rise in myelinated areas in nerve fibers surrounding oligodendroglial cells in CTX, HC and CC by 70.7%, 71% and 58%, respectively, compared to CPZ-RE group. While CPZ-RE + FIS group showed a significant difference from NC-9w group in CTX, it showed a non-significant difference in HC and CC (Fig. 4). Supplementary Figure 3 (Photomicrographs of CTX, HC and CC stained with LFB in 400x and 200x magnifications) is linked in the supplementary material.

**Fig. 5:**
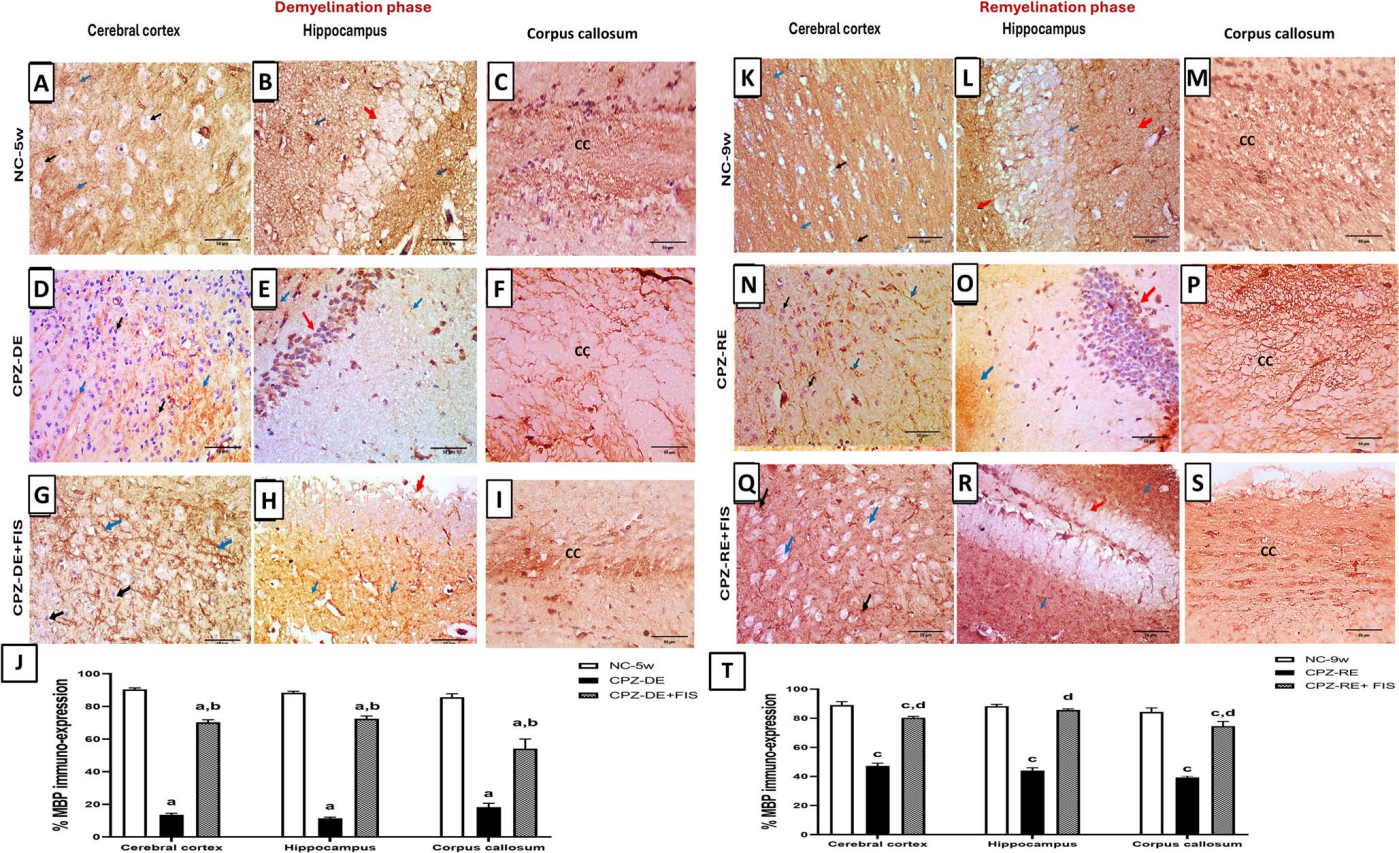
MBP Immunohistochemical analysis. (**A**-**I**,** K-S**): Photomicrographs of brain sections showing cerebral cortex, hippocampus and corpus callosum immunostained with MBP antibody, 400x magnification, scale bar 50 μm.(**J**,** T**): Statistical analysis of % MBP expression in demyelination and remyelination stages. For CTX, black arrow (oligodendroglial cells) and blue arrow (nerve fibers). For HC, red arrow (neurons) and blue arrow (nerve fibers). Data shown as mean ± SD (*n* = 3). Significance at *p* < 0.05; a: significant vs. NC-5w group, b: significant vs. CPZ-DE group, c: significant vs. NC-9w group, d: significant vs. CPZ-RE group. NC-5w: 5 week-normal control group; NC-9w: 9 week-normal control group; CPZ-DE: Cuprizone-demyelination group; CPZ-DE + FIS: Cuprizone-demyelination + Fisetin group; CPZ-RE: Cuprizone-remyelination group; CPZ-RE + FIS: Cuprizone-remyelination + Fisetin group; MBP: Myelin basic protein

De-and remyelination status were assessed by MBP immunostaining (Fig. 5). CPZ-DE group showed a significant (*p* < 0.0001) decline in MBP expression in CTX by 85%, HC by 87%, and CC by 79% relative to NC-5w mice. FIS treatment significantly (*p* < 0.0001) increased MBP expression in CTX by 5-folds, HC by 6-folds, and CC by 3-folds compared to CPZ-DE group. During remyelination, CPZ-RE + FIS group showed a significant (*p* < 0.0001) elevation in MBP expression by 70% in CTX, 94% in HC, and 89% in CC vs. CPZ-RE group. CPZ-RE + FIS group showed a significant difference from NC-9w group in CTX and CC but not in HC (Fig. 5). Supplementary Figure 4 (Photomicrographs of CTX, HC and CC stained with MBP antibody in 400x and 200x magnifications) is linked in the supplementary material.

### Effect of Fisetin on Astroglial Markers GFAP and Vimentin immuno-expression

**Fig. 6:**
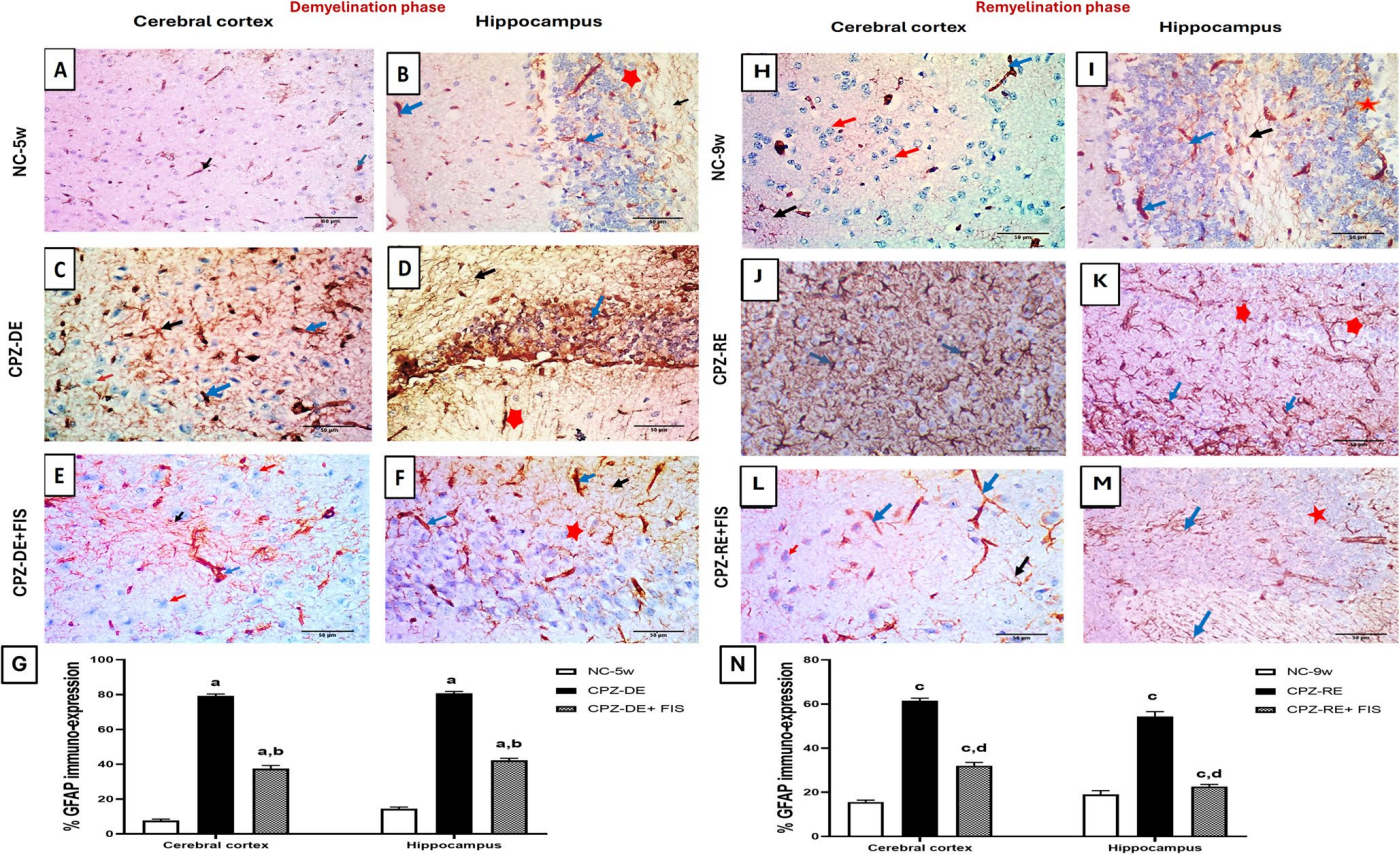
GFAP astroglial marker Immunohistochemical analysis. (**A**-**F**,** H**-**M**): Photomicrographs of brain sections showing cerebral cortex and hippocampus immunostained with GFAP antibody, 400x magnification, scale bar 50 μm.(**G**,** N**): Statistical analysis of % GFAP expression in demyelination and remyelination stages. For CTX, blue arrow (astrocytic cells), black arrow (nerve fibers) and red arrow (oligodendroglial cells). For HC, blue arrow (astrocytic cells), black arrow (nerve fibers) and red star (neurons). Data shown as mean ± SD (*n* = 3). Significance at *p* < 0.05; a: significant vs. NC-5w group, b: significant vs. CPZ-DE group, c: significant vs. NC-9w group, d: significant vs. CPZ-RE group. NC-5w: 5 week-normal control group; NC-9w: 9 week-normal control group; CPZ-DE: Cuprizone-demyelination group; CPZ-DE + FIS: Cuprizone-demyelination + Fisetin group; CPZ-RE: Cuprizone-remyelination group; CPZ-RE + FIS: Cuprizone-remyelination + Fisetin group; GFAP: Glial fibrillary acidic protein

CPZ diet significantly (*p* < 0.0001) increased GFAP immuno-expression in astrocytic cells and nerve fibers of CTX and HC by 10-folds and 6-folds vs. NC-5w group (Fig. 6). FIS treatment significantly (*p* < 0.0001) reduced GFAP immuno-expression in nerve fibers and astrocytic cells in CTX by 52%, and HC by 47%, compared to CPZ-DE group. During remyelination, CPZ-RE + FIS group significantly (*p* < 0.0001) reduced GFAP immuno-expression in CTX by 46%, and HC by 59%, vs. CPZ-RE group. GFAP immuno-expression in both regions of CPZ-RE + FIS group remained significantly elevated above NC-9w group values (Fig. 6). Supplementary Figure 5 (Photomicrographs of CTX and HC stained with GFAP antibody in 400x and 200x magnifications) is linked in supplementary material.

**Fig. 7:**
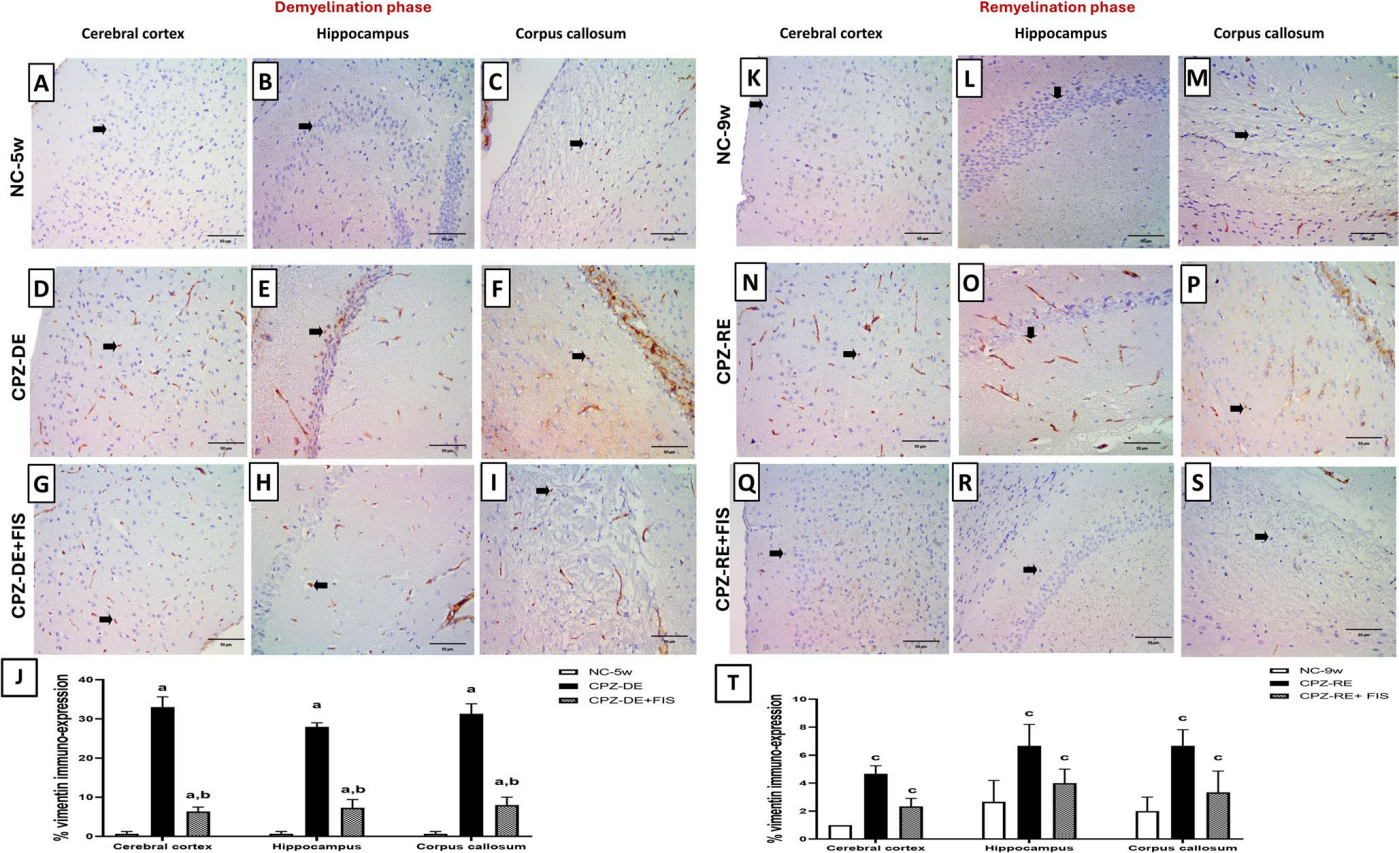
Vimentin astroglial marker Immunohistochemical analysis.(**A-I**,** K-S**): Photomicrographs of brain sections showing cerebral cortex, hippocampus and corpus callosum immunostained with vimentin antibody, 400x magnification, scale bar 50 μm.(**J**,** T**): Statistical analysis of % vimentin expression in demyelination and remyelination stages. For CTX, HC, and CC black arrow (neuroglia). Data shown as mean ± SD (*n* = 3). Significance at *p* < 0.05; a: significant vs. NC-5w group, b: significant vs. CPZ-DE group, c: significant vs. NC-9w group, d: significant vs. CPZ-RE group. NC-5w: 5 week-normal control group; NC-9w: 9 week-normal control group; CPZ-DE: Cuprizone-demyelination group; CPZ-DE + FIS: Cuprizone-demyelination + Fisetin group; CPZ-RE: Cuprizone-remyelination group; CPZ-RE + FIS: Cuprizone-remyelination + Fisetin group

CPZ-intoxication significantly (*p* < 0.0001) increased vimentin immuno-expression in neuroglia of CTX by 49-folds, HC by 42-folds and CC by 46-folds vs. NC-5w group (Fig. 7). FIS treatment significantly (*p* < 0.0001) diminished vimentin immuno-expression in neuroglia of CTX by 80%, HC by 73%, and CC by 74.4% compared to CPZ-DE group. During remyelination, CPZ-RE + FIS group significantly (*p* = 0.0187) lowered vimentin immuno-expression in CTX by 50%, (*p* = 0.0085) in HC by 40% and (*p* = 0.0017) in CC by 50% vs. CPZ-RE group. Vimentin immuno-expression in the three regions (CTX, HC, CC) sections of CPZ-RE + FIS group showed non-significant difference from NC-9w group (Fig. 7). Supplementary Figure 6 (Photomicrographs of CTX, HC and CC stained with vimentin antibody in 400x and 200x magnifications) is linked in the supplementary material.

### Effect of Fisetin on non-heme Iron Deposition

**Fig. 8:**
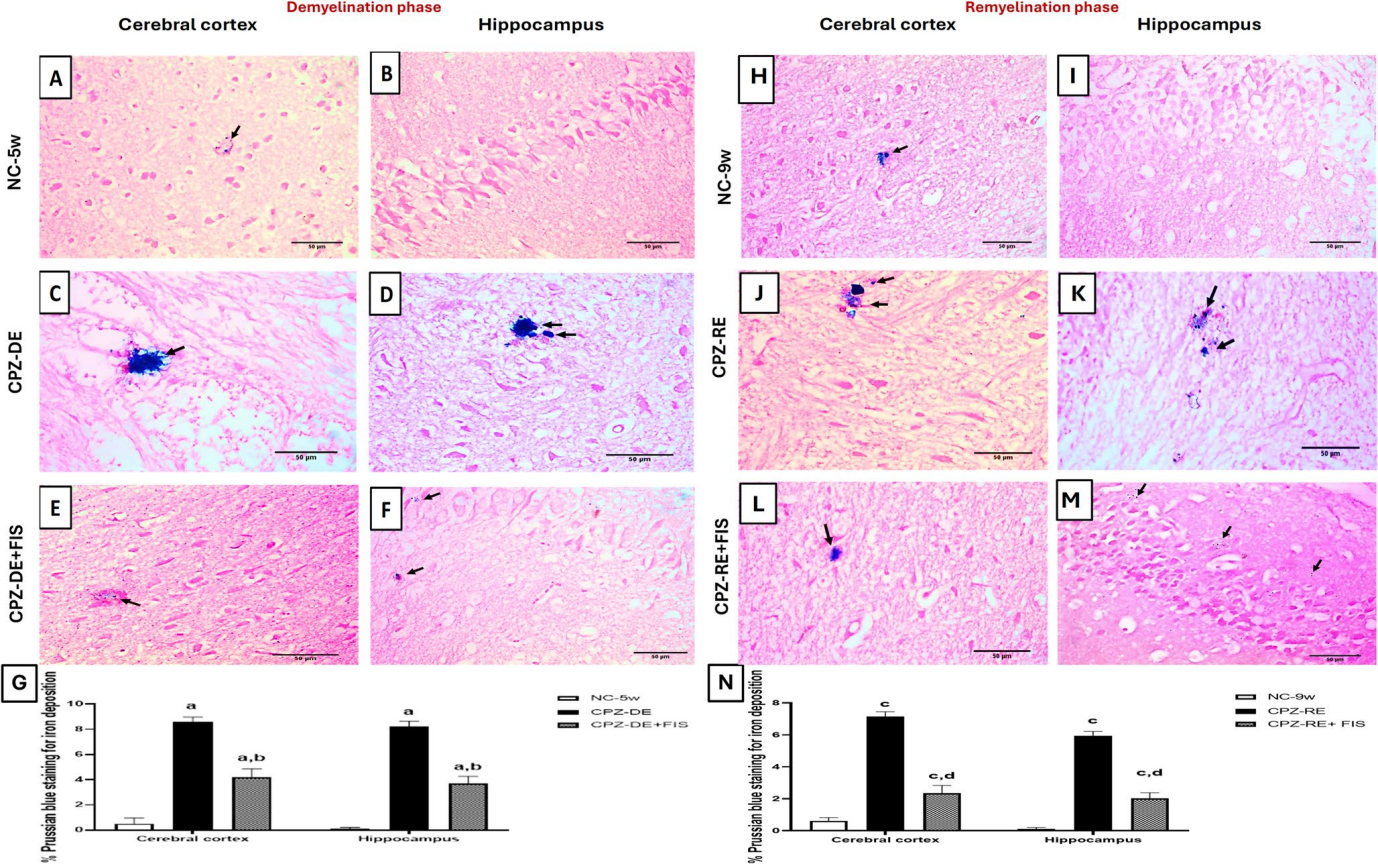
Effect of fisetin on non-heme iron deposition. (**A-F**,** H-M**): Photomicrographs of brain sections showing the cerebral cortex and hippocampus of groups stained with Perl’s Prussian blue, magnification 400x, scale bar 50 μm. (**G**,** N**): Statistical analysis of % non-heme iron deposition in demyelination and remyelination stages. Prussian blue stained iron deposition (black arrows). Data shown as mean ± SD (*n* = 3). Significance at *p* < 0.05; a: significant vs. NC-5w, b: significant vs. CPZ-DE, c: significant vs. NC-9w, d: significant vs. CPZ-RE. NC-5w: 5 week-normal control; NC-9w: 9 week-normal control; CPZ-DE: Cuprizone-demyelination; CPZ-DE + FIS: Cuprizone-demyelination + Fisetin; CPZ-RE: Cuprizone-remyelination; CPZ-RE + FIS: Cuprizone-remyelination + Fisetin

CPZ-DE group showed a significant (*p* < 0.0001) increase in iron deposits in CTX and HC by 17-fold and 41-fold vs. NC-5w group (Fig. 8). FIS administration significantly (*p* < 0.0001) reduced iron deposits in CTX and HC by 51% and 55% compared to CPZ-DE group. For remyelination groups, CPZ-RE + FIS group showed a significant (*p* < 0.0001) reduction in iron deposits in both regions by 67% and 65% vs. CPZ-RE group. Iron deposition in both regions of CPZ-RE + FIS group remained significantly higher than NC-9w group values (Fig. 8).

## Discussion

Multiple sclerosis (MS), the most prevalent disease to cause myelin loss, is one of the primary etiologies of long-term neurological dysfunction and disability in young adults, mainly due to insufficient effective remyelination in the adult human brain (Samy et al. 2023). Remyelination is an essential approach for the treatment of MS, as it is intimately connected to the repair of neuronal function and the mitigation of clinical disability (Cunniffe and Coles 2021). Presently, there is no efficacious therapeutic intervention for remyelination. Consequently, alternative MS therapies are nevertheless needed to foster remyelination and repair which in turn promote improvement in disease clinical symptoms and prevent neurodegeneration.

Evidence indicates that atypical stimulation of the ferroptosis pathway is present in neuroinflammatory diseases, including MS, in both patients and animal models. Additionally, ferroptosis markers may serve as valuable diagnostic tools in MS patients (Van San et al. 2023; Song et al. 2023). Ferroptosis has been implicated in the promotion of oligodendrocyte death and subsequent demyelination (Li et al. 2022). It has been assumed that ferroptosis triggered by induction of NCOA4-mediated ferritinophagy is a significant factor in the pathogenesis of neurodegenerative diseases (del Quiles and Mancias 2019)and ferroptosis inhibitors showed beneficial effects in CPZ demyelination model and a chronic experimental autoimmune encephalomyelitis (EAE) model of MS (Jhelum et al. 2020, 2023).

The present work intended to assess the neuroprotective and remyelinating effects of FIS in mice with CPZ-induced demyelination and its influence on modulating ferroptosis pathway which is interconnected to oxidative stress as a detrimental cell death modality in MS pathology. In the current study, CPZ consumption elicited behavioral and motor anomalies as established by tail suspension test and inverted screen grip strength test in both CPZ-DE and CPZ-RE groups compared to normal controls. It was previously established that behavioral and motor dysfunction coincided with the demyelination in the murine brain in distinct regions including CTX, HC and CC (Xue et al. 2022; Dupree et al. 2022; Leo and Kipp 2022). At the demyelination phase, our study showed that treatment with FIS significantly improved behavioral and motor defects, indicating the mitigation of CPZ-induced neurobehavioral and locomotor deficits. Meanwhile, during remyelination, FIS significantly improved behavioral disturbance to near normal values, but motor disturbance recovery wasn’t of significant value. In agreement with our results, FIS has exhibited neuroprotective effects on neurobehavioral and locomotor abnormalities in animal models of Alzheimer’s disease (AD), Parkinson’s disease (PD), and depression (Yu et al. 2016; Choubey et al. 2019; Alikatte et al. 2021).

In addition, the CPZ intoxication in our study caused neuronal injury evidenced by pyknotic oligodendroglial cells surrounded by highly edematous stroma in CTX, necrotic neurons with dense pyknotic nuclei surrounded by markedly degenerated edematous stroma in HC and degenerated nerve fibers and pyknotic nuclei in corpus callosum. CPZ cessation allowed partial restoration of normal oligodendroglial cells and packed neurons with less pyknotic nuclei surrounded by moderate edematous stroma. These pathological changes were previously described by (El Sharouny et al. 2022). Herein, FIS treatment was effective in restoring normal oligodendroglial cells surrounded by mild edematous stroma in CTX and densely packed neurons with vesicular nuclei as well as fewer degenerated neurons and nerve fibers with pyknotic nuclei in HC and CC during both de- and remyelination phases. These results indicate the promising potential of FIS treatment in alleviating neuronal damage inflicted during progressive MS.

In the current study, CPZ-DE and CPZ-RE groups demonstrated a marked upregulation in the mRNA expression of both NCOA4 and TfR1 and increase in TfR1 concentration compared to controls while there was a downregulation in FTH1 mRNA expression and decrease in ferritin concentration. The decreased FTH1 expression and ferritin protein level could be attributed to the increase in NCOA4 expression, a gene that codes for a cargo receptor that attaches to cytosolic ferritin and facilitates its transport to autophagosomes for degradation (ferritniophagy). Moreover, the increase in TfR1 expression and protein levels suggests an increase in iron cellular uptake as TfR1 attaches to transferrin-bound ferric iron, which is taken up by endosomes, and iron is released into the cytosol. This dysregulation in the iron uptake, storage and efflux leads to the increase in labile active iron, more non-heme iron deposits and subsequently lipid peroxidation and ferroptosis as described by (Jhelum et al. 2020). Our results were further supported by histological staining of non-heme iron deposits by Perl’s Prussian blue stain which revealed that CPZ-DE and CPZ-RE groups demonstrated a significant increase in iron deposition compared to controls indicating irregular iron accumulation in HC and CTX in MS animal model. Our study showed that the mRNA expressions of NCOA4 and TfR1, and TfR1 protein level in the brain tissues of mice treated with FIS were significantly downregulated, while FTH1 mRNA expression and ferritin level were significantly upregulated, and non-heme iron deposition was diminished during de-and remyelination phases compared to CPZ-DE and CPZ-RE groups. These results indicated that the neuroprotective effect of FIS during CPZ induced demyelination, and recovery could be linked to suppression of ferroptosis and ferritinophagy. In accordance with our results, recent studies on FIS have demonstrated anti-ferroptotic properties possibly through SIRT1/Nrf2 signaling activation and ACSL4-mediated ferroptosis inhibition (Li et al. 2022; Wang et al. 2023).

Oligodendrocytes (OLs) exhibit heightened vulnerability to oxidative stress, attributable to their elevated metabolic demand and limited antioxidant capacity due to OLs exhibiting low levels of the antioxidant glutathione. Therefore, previous research suggested that oxidative stress significantly influences the pathogenesis of MS (Spaas et al. 2021). GPX4 is a redox enzyme related to GSH which has the ability to suppress ferroptosis *via *mitigation of lipid peroxidation, a crucial hallmark of ferroptosis. GPX4 is imperative for the reduction of reactive aldehydes (PUFAs-OOH) to the corresponding alcohol form (PUFAs-OH), hence decreasing ROS levels (Xu et al. 2021). In the present study, CPZ ingestion in CPZ-DE and CPZ-RE groups significantly increased MDA mice brain levels and significantly decreased the antioxidant GPX4 content in mice brain tissues compared to controls, thereby triggering oxidative stress, lipid peroxidation and ferroptosis. These findings align with earlier studies that identify ROS as mediators of demyelination and neuronal injury in both MS and its experimental models (Ghiasian et al. 2019; Namazi et al. 2022). In addition, our results showed that FIS treatment significantly lessened MDA concentration and significantly augmented the GPX4 concentration in mice brain during de-and remyelination, emphasizing the antioxidant effect of FIS during CPZ-induced demyelination and fostering remyelination. FIS has been previously documented to exhibit antioxidant potential in various diseases and neurodegenerative animal models possibly through the AMPK/PI3K/AKT-mediated Nrf2 antioxidant defense mechanism (Wang et al. 2023; Moustafa et al. 2024). Therefore, we theorized that the advantageous effects of FIS in treatment of CPZ-induced demyelination and enhancement of remyelination might be associated with the modulation of ferroptosis, NCOA4-mediated ferritinophagy pathway, and antioxidant activity.

One defining characteristic of MS is the degeneration of the myelin sheath. MBP is an imperative component of mature myelin that helps build its densely multilayered structure. It is found throughout the myelin sheath and is exclusively expressed by OLs, making MBP an excellent marker for demonstrating remyelination processes (Elbaz et al. 2018). Our study demonstrated significant myelin injury in CPZ-fed mice, evidenced by a decline in the percentage of MBP immuno-expression and a reduction in the percentage of myelinated areas in LFB-stained sections of the CTX, HC and CC compared to control mice. Evidence indicates that demyelination and a reduction in MBP immuno-expression and OLs occur during the initial 3–5 weeks of CPZ intoxication, followed by spontaneous remyelination upon CPZ withdrawal (Liu et al. 2020; Ai et al. 2022), which is consistent with our findings. Our results indicated that the quantitative analysis of MBP immuno-expression and the percentage of myelinated areas in LFB-stained CTX, HC and CC sections were significantly augmented in the FIS-treated groups in comparison with the CPZ groups during de- and remyelination. Notably, the HC sections’ results reached almost normal control levels during remyelination indicating the capacity of FIS to suppress demyelination and its promising potential to stimulate remyelination.

In MS lesions, activated astrocytes secrete pro-inflammatory cytokines, which sequentially attract immune cells, hinder the maturation of oligodendrocyte precursor cells (OPCs), and contribute to the formation of a glial scar that obstructs the migration of OPCs into demyelinated regions (Correale and Farez 2015). GFAP serves as a key marker for astrocytes; however, it is variably expressed across distinct brain regions (Castillo-Rodriguez et al. 2022). Our study revealed that the immuno-expression of GFAP in the CTX and HC significantly increased after CPZ intoxication compared to control mice indicating active astrogliosis in CPZ induced demyelination which is then followed by mitigated astrogliosis after CPZ withdrawal as demonstrated in previous studies (Dupree et al. 2022; Castillo-Rodriguez et al. 2022). Moreover, FIS treated groups exhibited a significant decline in the expression of GFAP in the CTX and HC relative to CPZ groups during demyelination and recovery. This suggests that attenuation of astrocyte activation by FIS therapy is considered advantageous for myelin regeneration and repair. In accordance with our results, it was reported that FIS effectively attenuated gliosis and astrocyte activation in animal models of dementia and LPS-induced neurotoxicity (Currais et al. 2018; Ahmad et al. 2019).

Vimentin is a principal type III intermediate filament protein that plays a focal role in neuronal structural development and functional dynamics and a recognized marker of activated microglia and macrophages. Vimentin is a non-specific marker for microglia but under neuroinflammatory conditions, upregulated vimentin is essential for microglial activation and cellular plasticity, therefore lack of vimentin confers neuroprotection in vivo, while astrocytic vimentin along with GFAP similarly orchestrate cytokine signaling, reactive gliosis and glial scar formation (Jurga et al. 2020). Our study revealed that the immuno-expression of vimentin in the CTX, HC and CC significantly increased after CPZ diet compared to control mice indicating active astrocytes and microglia in CPZ induced demyelination which is then followed by lessened astrogliosis after CPZ withdrawal as established in previous research (Ai et al. 2022). Furthermore, FIS treatment resulted in a significant decrease in the expression of vimentin in the CTX, HC and CC relative to CPZ only groups during demyelination and remyelination groups showing near normal levels. This observation implies that attenuation of astroglial activation in demyelinating disorders like MS through diminishing vimentin expression by FIS therapy is considered beneficial for axonal remyelination.

Oligodendrocytes are the cells responsible for CNS myelination, originating from OPCs which function as proliferative adult progenitor cells differentiating into mature OLs to generate myelin to wrap tightly around axons (Kuhn et al. 2019). Olig-1 is a central regulator of OPCs differentiation, and subsequent myelination primarily in brain development and repair. Olig-1 regulates the transcription of key myelin-specific genes, including MBP, PLP1, and MAG, while suppressing the expression of GFAP at the molecular level (Dai et al. 2015). In the present work, Olig-1 gene expression in mice brain was downregulated compared to controls following CPZ intoxication in CPZ-DE and CPZ-RE groups, demonstrating that maturation of OPCs and subsequent myelination of axons are suppressed in the CPZ-induced demyelination model, which agrees with studies by (Semnani et al. 2016; Ghaiad et al. 2017). Our results also demonstrated FIS ability to induce OPCs proliferation and differentiation in brain tissue as indicated by upregulated Olig-1 gene expression in FIS treated group compared to CPZ-fed group during demyelination, suggesting FIS potential to halt demyelination through modulating Olig-1 expression.

Neuroinflammation contributes significantly to the pathogenesis and progression of MS (Al-Badri and Castorina 2018). IL-1β is one of the pro-inflammatory cytokines which is highly expressed in severe stages of MS triggering autoimmunity and neuroinflammation-dependent CNS damage (Mantovani et al. 2019). Our study detected a significant boost in brain concentrations of IL-1β in both CPZ-DE and CPZ-RE groups compared to control mice which provides indication of neuroinflammation induced by CPZ intoxication. Furthermore, FIS treatment alleviated neuroinflammation by significantly attenuating the brain IL-1β concentrations in CPZ-fed mice during de-and remyelination with returning to near normal values in CPZ-RE + FIS group, signifying the anti-inflammatory potential of FIS to attenuate demyelination and promote remyelination, which was supported by the previously demonstrated anti-inflammatory properties of FIS that can be attributed to FIS ability to suppress MCP 1, iNOS and IL-1R/TLR axis (Hada et al. 2021; Khatoon et al. 2021). While our findings emphasize fisetin’s neuroprotective potential in the cuprizone model of MS, the study’s single-batch design constrains reproducibility and necessitates independent validation across additional animal cohorts. The exclusive use of an 80 mg/kg dose precludes dose–response characterization and pharmacokinetic–pharmacodynamic correlations. Although we demonstrate ferroptosis and neuroinflammation modulation, we have not yet explored overlapping pathways such as apoptosis or autophagy. Behavioral outcomes focus solely on locomotor metrics; future work should integrate cognitive, affective, and sensory assays to report more in-depth functional recovery. Future research should focus on replicating the results, optimizing dosing, extending timelines, and broadening mechanistic and behavioral assessments to establish fisetin as promising MS therapy.

## Conclusion

Our study was the first to demonstrate that FIS exerted a neuroprotective impact in mice with CPZ-induced demyelination through improving neurobehavioral defects, alleviating neuronal injury, mitigating oxidative stress and neuroinflammation, as well as promoting remyelination. The neuroprotective effect of FIS was proved to be mediated, in part, by suppression of ferroptosis signaling pathway and its link to ferritinophagy NCOA4 cargo. Thus, FIS could be a promising adjuvant therapy for improving the therapeutic outcomes in MS and other demyelinating disorders and modulating ferroptosis could be a novel neuroprotective intervention for the treatment of neurodegenerative disorders which warrants further investigations in clinical settings.

## Supplementary Information

Below is the link to the electronic supplementary material.

Supplementary Material 1

Supplementary figure 2 (PNG 4.25 MB)

High Resolution Image (TIF 60.1 MB)

Supplementary figure 3 (PNG 4.09 MB)

High Resolution Image (TIF 51.8 MB)

Supplementary figure 4 (PNG 4.45 MB)

High Resolution Image (TIF 52.5 MB)

Supplementary figure 5 (PNG 3.52 MB)

High Resolution Image (TIF 42.5 MB)

Supplementary figure 6 (PNG 3.91 MB)

High Resolution Image (TIF 43.6 MB)

## Data Availability

All the data generated and/or analyzed during performing this current study are included in this article. However, there is no restriction on the availability of materials and data from the corresponding author on reasonable request.

## Abbreviations

4 HNE: 4-Hydroxy-2-Nonenal
ACSL-4: Acyl-CoA synthetase long-chain family member 4
AD: Alzheimer’s Disease
AKT: Protein Kinase B
AMPK: AMP-activated protein kinase
ANOVA: Analysis of Variance
cDNA: Complementary DNA
CTX: Cerebral cortex
CNS: Central Nervous System
CPZ: Cuprizone
CPZ-DE: Cuprizone-Demyelination
CPZ-DE + FIS: Cuprizone-Demyelination + Fisetin
CPZ-RE: Cuprizone-Remyelination
CPZ-RE + FIS: Cuprizone-Remyelination + Fisetin
Ct: Threshold cycle
DAB: Diaminobenzidine
DMTs: Disease Modifying Therapies
EAE: Experimental Autoimmune Encephalomyelitis
F: Forward primer
FDA: Food and drug administration
FIS: Fisetin (3,3′,4′,7-tetrahydroxyflavone)
FTH1: Ferritin Heavy Chain 1
FTL: Ferritin Light Chain
GFAP: Glial Fibrillary Acidic Protein
GPX4: Glutathione Peroxidase 4
GSH: Glutathione
H&E: Hematoxylin and Eosin
HC: Hippocampus
IL-1β: Interleukin- 1 Beta
IL-1R: Interleukin-1 receptor
iNOS: inducible nitric oxide synthase
LFB: Luxol Fast Blue
LPO: Lipid Peroxidation
LSD: Least Significant Differences
MBP: Myelin Basic Protein
MCP-1: Monocyte chemoattractant protein-1
MDA: Malondialdehyde
MS: Multiple Sclerosis
Na CMC: Sodium Carboxymethyl Cellulose
NC: Normal Control
NCOA4: Nuclear Receptor Coactivator 4
NIH: National Institutes of Health
Nrf2: Nuclear factor erythroid 2-related factor 2
Olig-1: Oligodendrocyte Transcription Factor-1
OLs: Oligodendrocytes
PB: Prussian Blue
PBS: Phosphate Buffered Saline
PCR: Polymerase Chain Reaction
PD: Parkinson’s Disease
PI3K: Phosphoinositide 3-kinase
PUFAs: Polyunsaturated Fatty Acids
qPCR: Quantitative Polymerase Chain Reaction
qRT PCR: Quantitative Real Time Polymerase Chain Reaction
R: Reverse primer
ROS: Reactive oxygen species
RQ: Relative quantity
RRMS: Relapsing Remitting Multiple Sclerosis
SD: Standard Deviation
SPMS: Secondary Progressive Multiple Sclerosis
SYBR: SYBR Green
TBARS: Thiobarbituric Acid Reactive Substances
TfR1: Transferrin receptor 1
TLR: Toll-like receptor
TST: Tail suspension test
