# Supplementary material for: Fisetin Mitigates Ferroptosis and Promotes Remyelination in a Cuprizone Model of Multiple Sclerosis
Source: J Neuroimmune Pharmacol. 2025 Dec 9;20(1):108. doi: 10.1007/s11481-025-10260-z (PMC12685980; doi:10.1007/s11481-025-10260-z)
Supplement: Supplementary file 1 — Supplementary Material 1 [file 11481_2025_10260_MOESM1_ESM.docx]

**Supplementary figures’ captions and comments**

- **Suppl. to Figure 3: Representative photomicrographs of cerebral cortex, hippocampus and corpus callosum (H&E) 400x and 200x magnifications (A-R).**

(A, J): Cerebral cortex in NC-5w and NC-9w groups showing normal histological architecture of neocortex consisting of six layers; (1) molecular, (2) external granular, (3) external pyramidal, (4) internal granular, (5) internal pyramidal and (6) polymorphic cell layer. Intact pia matter was seen (thin arrow) (Magnification 200x). The 400x magnification photomicrographs showing intact pia matter (thin arrow), normal granular cells with rounded pale nuclei (yellow arrow), pyramidal cells (red arrow), neuroglial cells with small dark nuclei (blue arrow) surrounded by normal neuropil (*). Normal blood capillary was seen (bc). (B, K): Hippocampus in NC-5w and NC-9w groups showing normal histological architecture formed of cornu ammonis (CA) and dentate gyrus (DG) separated by a hippocampal sulcus (HS) (Magnification 200x). The 400x magnification photomicrographs showing CA1 region with well-defined three layers; polymorphic layer (POL), pyramidal cell layer (PCL) and molecular layer (ML). PCL exhibits closely packed cell bodies of the pyramidal neurons (bifid black arrow) that are regularly arranged in 3 to 4 rows and appear small with vesicular nuclei, prominent nucleoli and scanty cytoplasm. The POL and ML display deeply (dg) and lightly (lg) stained nuclei of glial cells with normal blood capillaries (BC). (C, L): Corpus callosum in NC-5w and NC-9w groups showing normal histological architecture formed of densely packed axons (CC) next to cerebral cortex (C). parts of hippocampus (HP) and choroid plexus (CP) were seen (Magnification 200x). The 400x photomicrographs showing densely packed axons (bifid green arrow), small dense oligodendrocyte arranged in short chains (bifid white arrows), deeply stained nuclei of glial cells (g).

(D) Cerebral cortex of CPZ-DE group showing small deeply stained nuclei of different layers surrounded by perinuclear halo (arrowheads) and dilated congested blood vessels (BV). Intact pia matter was seen (thin arrow) (Magnification 200x). The 400x magnification photomicrograph showing small deeply stained nuclei of granular cells (yellow arrow) and pyramidal cells (red arrow) and neuroglial cells (blue arrow) with perinuclear halo surrounded by vacuolated neuropil (*). Intact pia matter was seen (thin arrow). (E) Hippocampus of CPZ-DE group showing its different regions; CA1, CA2 and HS (Magnification 200x). The 400x magnification photomicrograph showing CA1 region with well-defined three layers; POL with vacuolated neuropil, decreased number of cell layers pyramidal cell (PCL) and molecular layer (ML), most of pyramidal neurons with pyknotic nuclei (pn), perinuclear halo around lightly stained nuclei (lg) and deeply stained nuclei of glial cells (dg). (F) Corpus callosum of CPZ-DE group showing disrupted histological architecture with degenerated nerve fibers (Magnification 200x). The 400x magnification photomicrograph showing loosely arranged degenerated axons (bifid green arrows) and decrease number of scattered small dense pyknotic nuclei of oligodendrocytes (bifid white arrows).

(G) Cerebral cortex of CPZ-DE-FIS group showing moderate improvement of histological architecture of neocortex. Most of neuronal cells were of pale stained nuclei but surrounded by perinuclear halo (red arrowhead) and normal neuropil (*). There was decreased neuronal density (white arrowhead). Intact pia matter (thin arrow) (Magnification 200x). The 400x magnification photomicrograph showing moderate improvement of histological architecture of neocortex. Most of neuronal cells were of pale stained nuclei but surrounded by perinuclear halo (red arrowhead, others revealed some stained pyknotic nuclei (black arrowhead) and normal neuropil (*). Intact pia matter (thin arrow). (H) Hippocampus of CPZ-DE-FIS group showing moderate improvement with its different regions; CA1, CA2 and HS (Magnification 200x). The 400x magnification of CA1 region of the previous photomicrograph showing well-defined three layers POL, PCL and molecular ML. PCL exhibits closely packed cell bodies of the pyramidal neurons (bifid black arrow) that are regularly arranged in 3 to 4 rows and appear small with vesicular nuclei, prominent nucleoli and scanty cytoplasm. Few cells were of pyknotic nuclei (pn). The POL layer display deeply (dg) and lightly (lg) stained nuclei of glial cells but surrounded by perinuclear halo. (I) Corpus callosum of CPZ-DE-FIS group showing moderate improvement of corpus callosum formed of densely packed axons (CC) between cerebral cortex (C) and hippocampus (HP) (Magnification 200x). The 400x magnification photomicrograph showing densely packed axons (bifid green arrow), small dense nuclei of oligodendrocyte arranged in short chains (bifid white arrows), deeply stained nuclei of glial cells (g).

(M) Cerebral cortex of CPZ-RE group showing mild improvement of histological architecture of neocortex including (1) molecular, (2) external granular, (3) external pyramidal, (4) internal granular, (5) internal pyramidal and (6) polymorphic cell layer. Intact pia matter (thin arrow) was seen (Magnification 200x). The 400x magnification photomicrograph showing few granular (yellow arrow) and pyramidal cells (red arrowhead) were normal pale stained nuclei while some pyramidal cells with shrunken pyknotic nuclei (red arrow) surrounded by vacuolated neuropil (*). (N) Hippocampus of CPZ-RE group showing its different regions; CA3 and CA4 and DG separated by HS (Magnification 200x).The 400x magnification photomicrograph of CA3 and 4 regions photomicrograph showing well-defined three layers; POL, PCL and ML, some normal pyramidal neurons (bifid black arrow), deeply stained nuclei of glial cells (dg) and blood capillaries (bc) shrunken pyknotic nuclei of pyramidal neurons (pn) and perinuclear halo around lightly stained nuclei of glial cells (lg). (O) Corpus callosum of CPZ-RE group showing mild improvement of histological architecture of corpus callosum (CC) (Magnification 200x). The 400x magnification photomicrograph showing mild improvement except for focal areas of degenerated axons (bifid green arrow), dilated blood capillary (BC). Increased number of small dense nuclei of oligodendrocytes (bifid white arrows).

(P) Cerebral cortex of CPZ-RE-FIS group showing marked improvement of histological architecture of neocortex more or less similar to normal control group consisting of six layers; (1) molecular, (2) external granular, (3) external pyramidal, (4) internal granular, (5) internal pyramidal and (6) polymorphic cell layer. Intact pia matter (thin arrow) and normal blood capillary (BC) were seen (Magnification 200x). The 400x magnification photomicrograph showing intact pia matter (thin arrow), normal granular cells with rounded pale nuclei (yellow arrow), pyramidal cells (red arrow), neuroglial cells with small dark nuclei (blue arrow) surrounded by normal neuropil (*) and normal blood capillary (BC). (Q) Hippocampus of CPZ-RE-FIS group showing marked improvement with its different regions; CA and DG separated by a HS. CA1, CA2 and CA3 regions were seen (Magnification 200x). The 400x magnification of CA 1 region showing well-defined three layers; POL, PCL and molecular layer ML. PCL exhibits closely packed cell bodies of the pyramidal neurons (bifid black arrow) that are regularly arranged in 3 to 4 rows and appear small with vesicular nuclei, prominent nucleoli and scanty cytoplasm. The POL and ML display deeply (dg) and lightly (lg) stained nuclei of glial cells similar to normal control group. (R) Corpus callosum of CPZ-RE-FIS group showing marked improvement of corpus callosum formed of densely packed axons (CC) between cerebral cortex (C) and hippocampus (HP) (Magnification 200x). The 400x magnification of the previous photomicrograph showing densely packed axons (bifid green arrow), small dense nuclei of oligodendrocyte arranged in short chains (bifid white arrows), and deeply stained nuclei of glial cells (g) more or less similar to normal control group. NC-5w: 5 week-normal control group; NC-9w: 9 week-normal control group; CPZ-DE: Cuprizone-demyelination group; CPZ-DE+FIS: Cuprizone-demyelination+Fisetin group; CPZ-RE: Cuprizone-remyelination group; CPZ-RE+FIS: Cuprizone-remyelination+Fisetin group

- **Suppl. to Figure 4: Effect of fisetin on brain myelination state by LFB staining. (A-R): Photomicrographs of brain sections showing cerebral cortex, hippocampus and corpus callosum of studied groups stained with luxol fast blue (LFB), 400x and 200x magnifications.** For cerebral cortex and hippocampus, and corpus callosum, blue arrow (neurons) and black arrow (nerve fibers). NC-5w: 5 week-normal control group; NC-9w: 9 week-normal control group; CPZ-DE: Cuprizone-demyelination group; CPZ-DE+FIS: Cuprizone-demyelination+Fisetin group; CPZ-RE: Cuprizone-remyelination group; CPZ-RE+FIS: Cuprizone-remyelination+Fisetin group; cornu ammonis 1,2,3 (CA1,2,3).
- **Suppl. to Figure 5: Effect of fisetin on brain myelination state by MBP immuno-staining. (A-R): Photomicrographs of brain sections showing cerebral cortex, hippocampus and corpus callosum of studied groups stained with MBP antibody, 400x and 200x magnifications.** For cerebral cortex and hippocampus, and corpus callosum blue arrow (neurons) and black arrow (nerve fibers). NC-5w: 5 week-normal control group; NC-9w: 9 week-normal control group; CPZ-DE: Cuprizone-demyelination group; CPZ-DE+FIS: Cuprizone-demyelination+Fisetin group; CPZ-RE: Cuprizone-remyelination group; CPZ-RE+FIS: Cuprizone-remyelination+Fisetin group; cornu ammonis 1,2,3 (CA1,2,3).
- **Suppl. to Figure 6: Effect of fisetin on** **astroglial GFAP immuno-staining. (A-L): Photomicrographs of brain sections showing cerebral cortex and hippocampus of studied groups stained with GFAP antibody, 400x and 200x magnifications.** For cerebral cortex and hippocampus, blue arrow (astrocytes) and black arrow (nerve fibers). NC-5w: 5 week-normal control group; NC-9w: 9 week-normal control group; CPZ-DE: Cuprizone-demyelination group; CPZ-DE+FIS: Cuprizone-demyelination+Fisetin group; CPZ-RE: Cuprizone-remyelination group; CPZ-RE+FIS: Cuprizone-remyelination+Fisetin group; cornu ammonis 1,2,3 (CA1,2,3).
- **Suppl. to Figure 7: Effect of fisetin on astroglial vimentin immuno-staining. (A-R): Photomicrographs of brain sections showing cerebral cortex, hippocampus and corpus callosum of studied groups stained with vimentin antibody, 400x and 200x magnifications.** For cerebral cortex, hippocampus and corpus callosum black arrow (neuroglia). NC-5w: 5 week-normal control group; NC-9w: 9 week-normal control group; CPZ-DE: Cuprizone-demyelination group; CPZ-DE+FIS: Cuprizone-demyelination+Fisetin group; CPZ-RE: Cuprizone-remyelination group; CPZ-RE+FIS: Cuprizone-remyelination+Fisetin group; cornu ammonis 1,2,3 (CA1,2,3)
