# Supplementary figures and images for: Fisetin Mitigates Ferroptosis and Promotes Remyelination in a Cuprizone Model of Multiple Sclerosis
Source: J Neuroimmune Pharmacol. 2025 Dec 9;20(1):108. doi: 10.1007/s11481-025-10260-z (PMC12685980; doi:10.1007/s11481-025-10260-z)

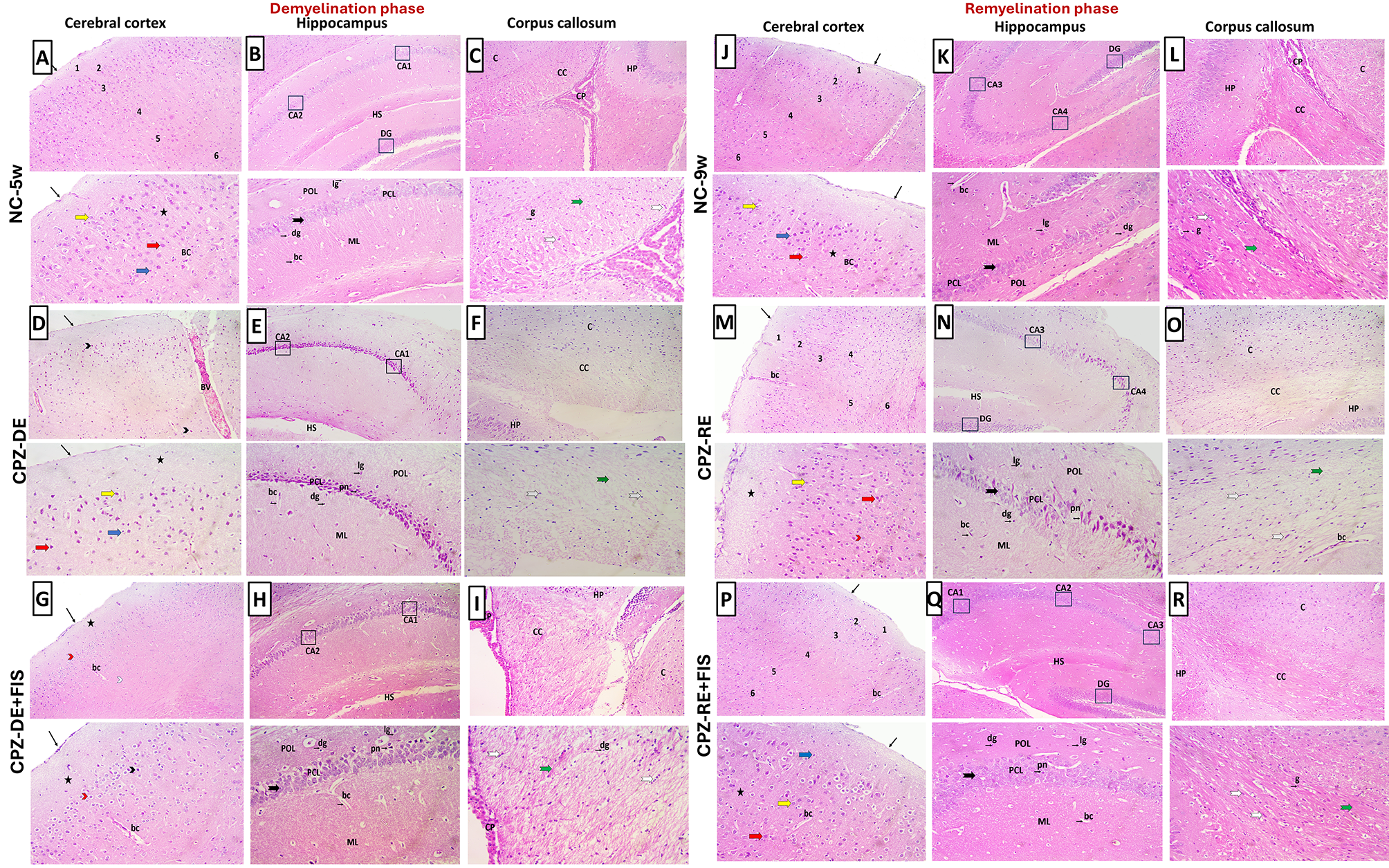

Supplement: Supplementary file 2 — (PNG 4.25 MB) [file 11481_2025_10260_Fig9_ESM.png]

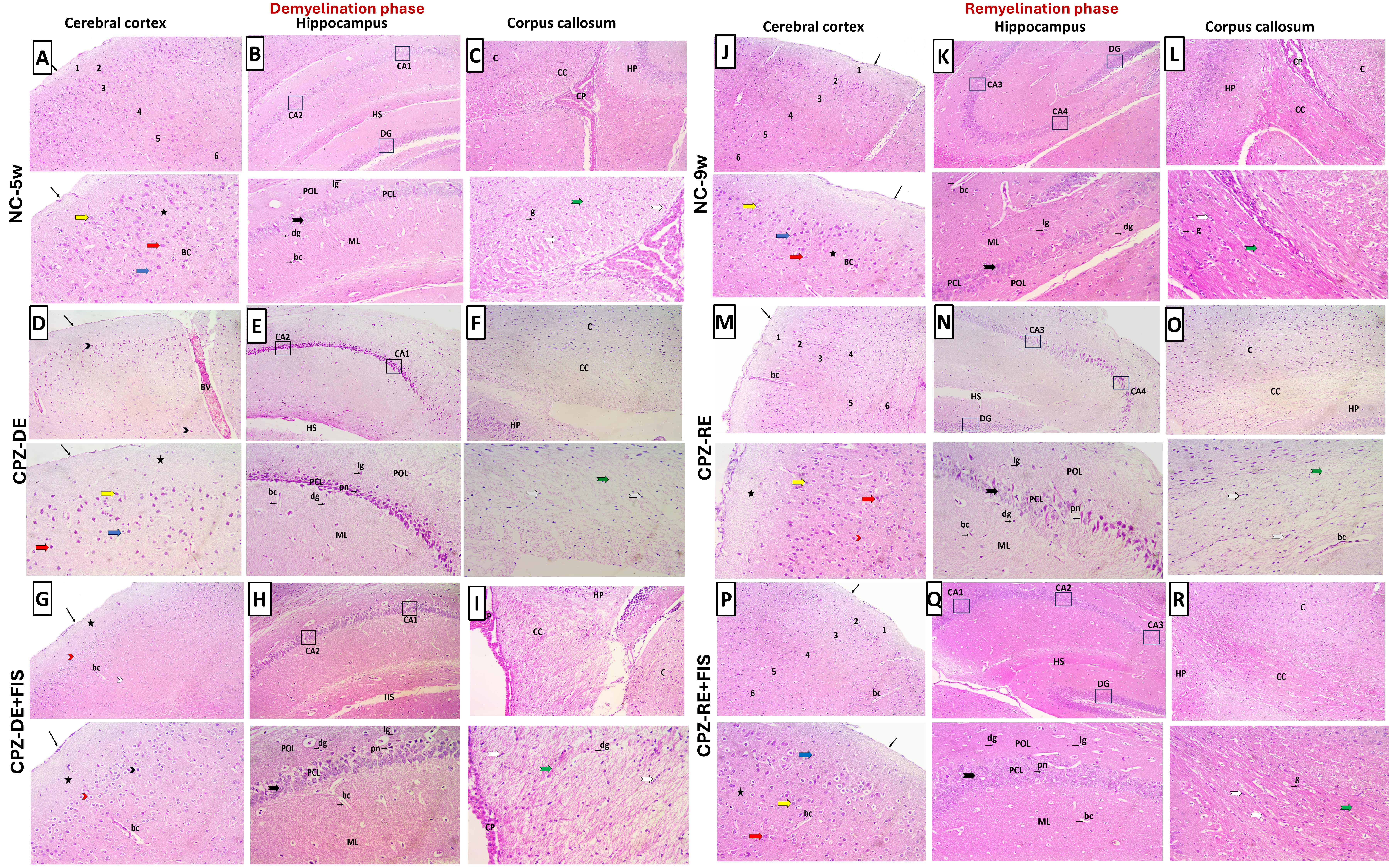

Supplement: Supplementary file 3 — High Resolution Image (TIF 60.1 MB) [file 11481_2025_10260_MOESM2_ESM.tif]

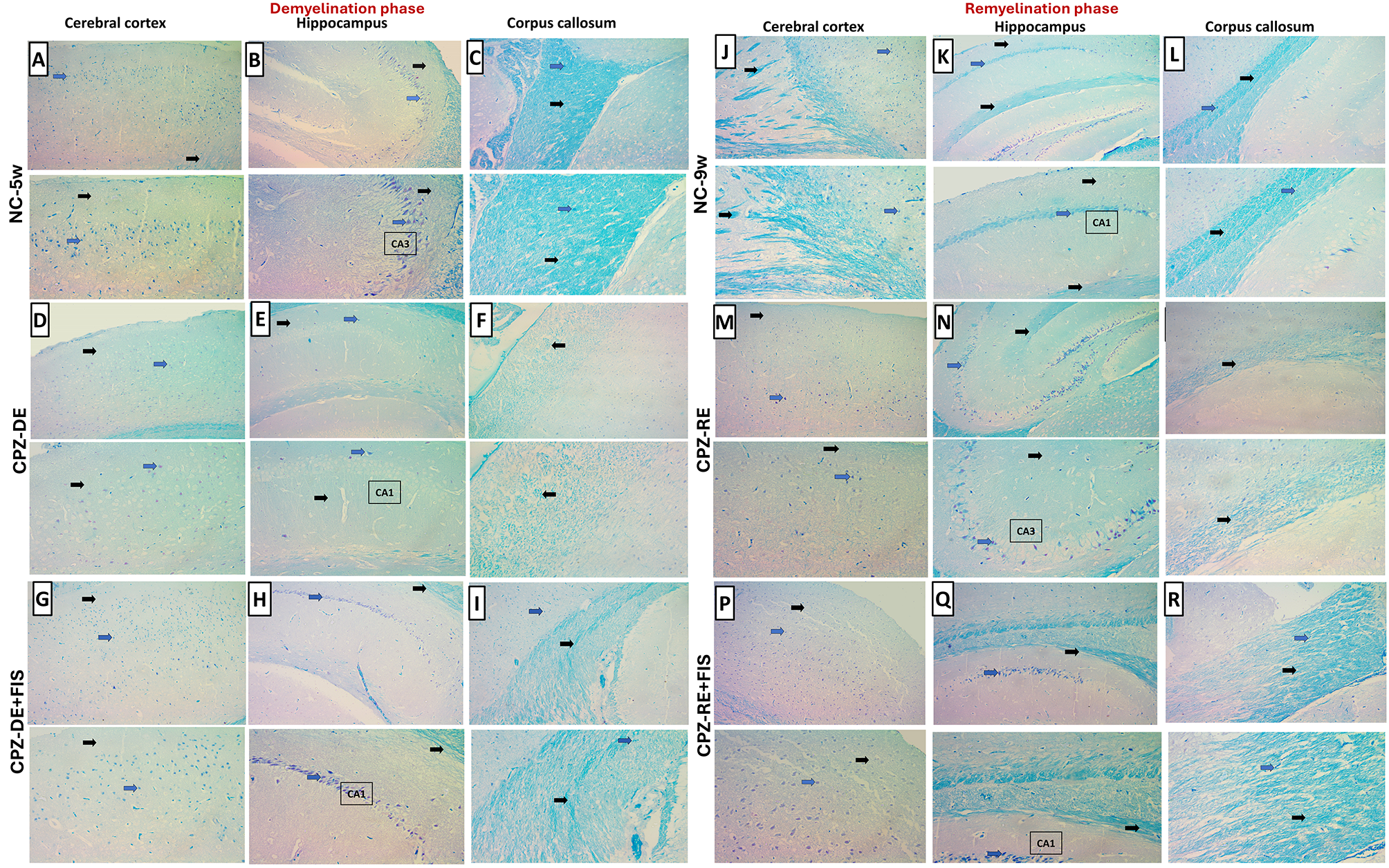

Supplement: Supplementary file 4 — (PNG 4.09 MB) [file 11481_2025_10260_Fig10_ESM.png]

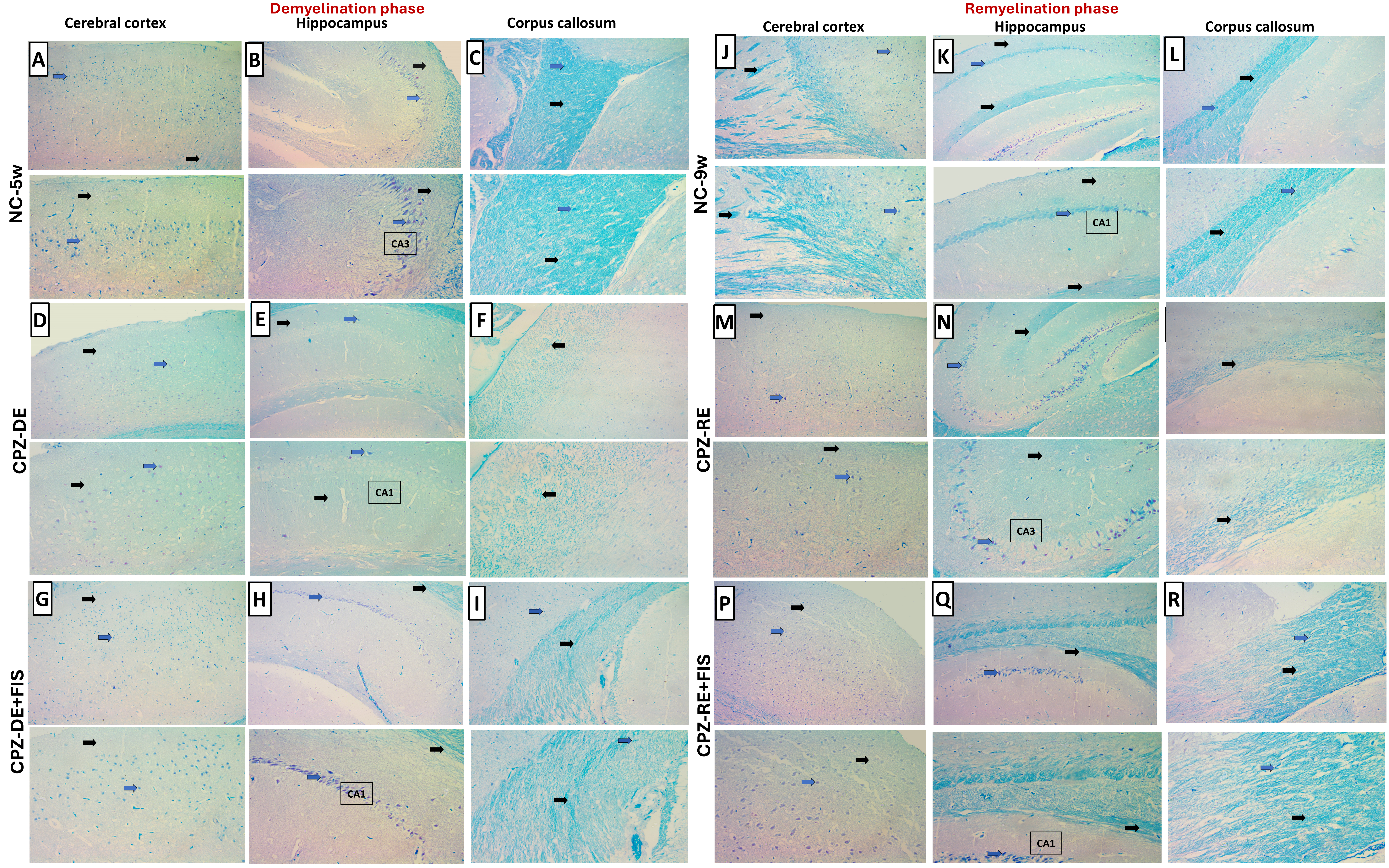

Supplement: Supplementary file 5 — High Resolution Image (TIF 51.8 MB) [file 11481_2025_10260_MOESM3_ESM.tif]

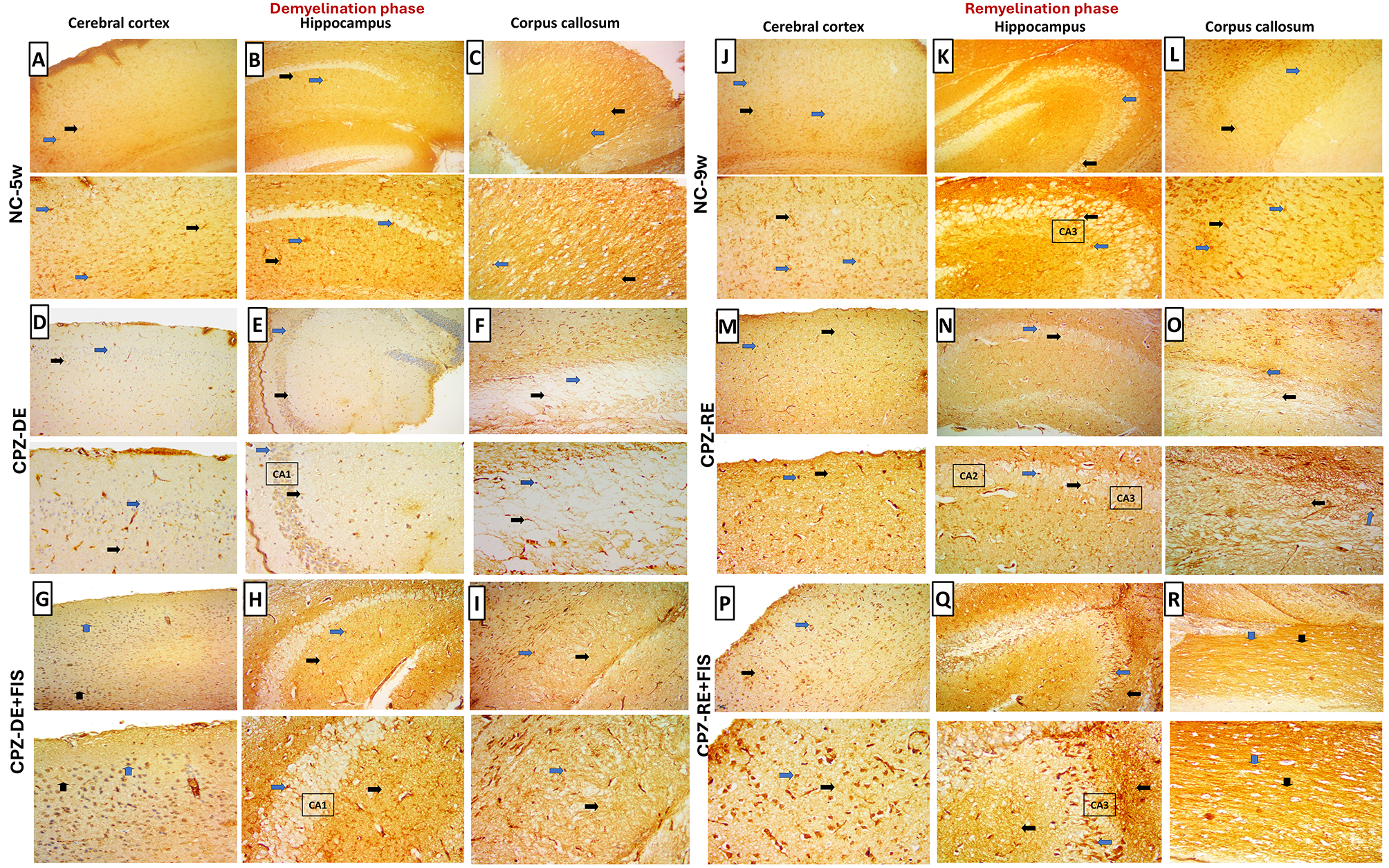

Supplement: Supplementary file 6 — (PNG 4.45 MB) [file 11481_2025_10260_Fig11_ESM.png]

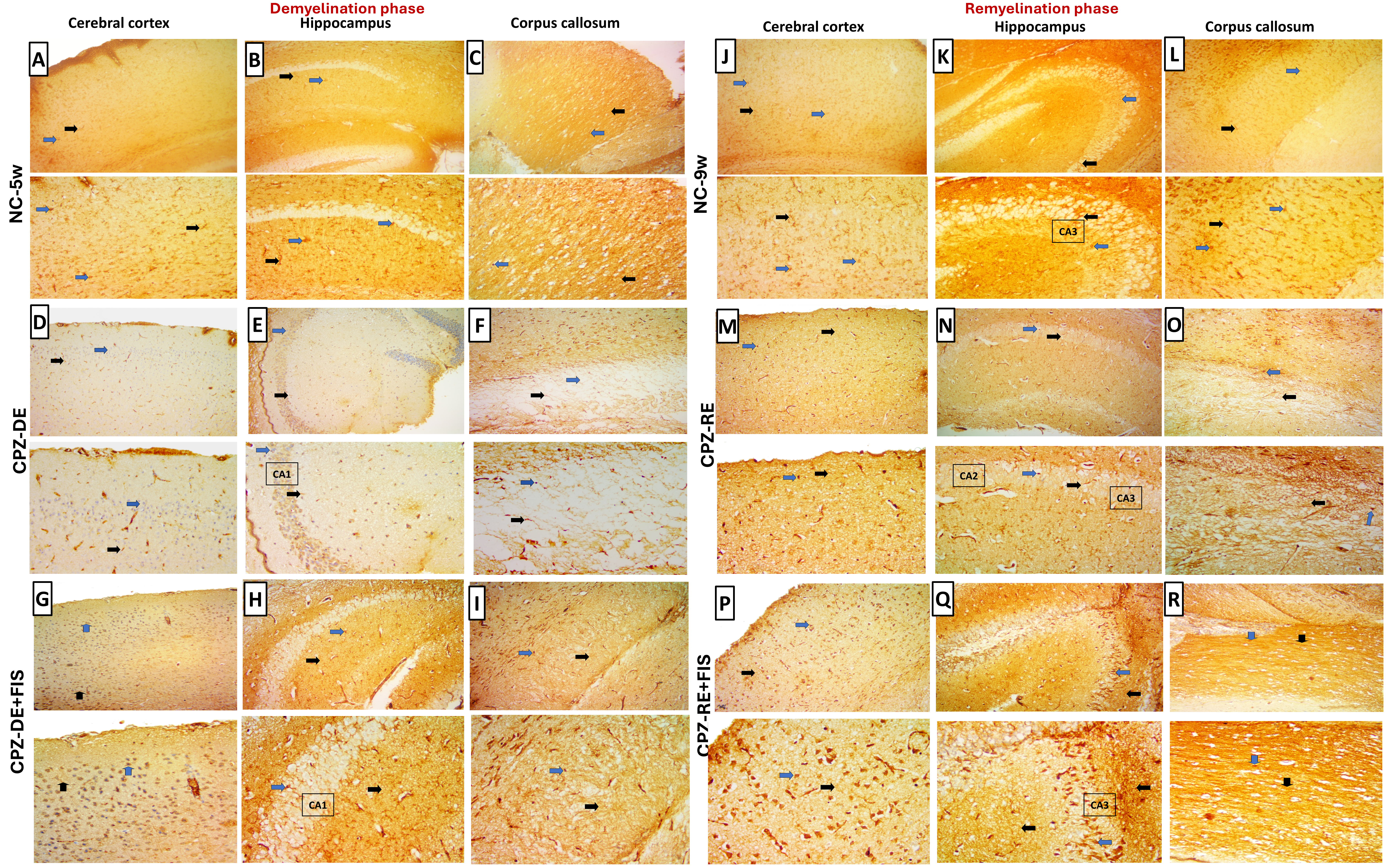

Supplement: Supplementary file 7 — High Resolution Image (TIF 52.5 MB) [file 11481_2025_10260_MOESM4_ESM.tif]

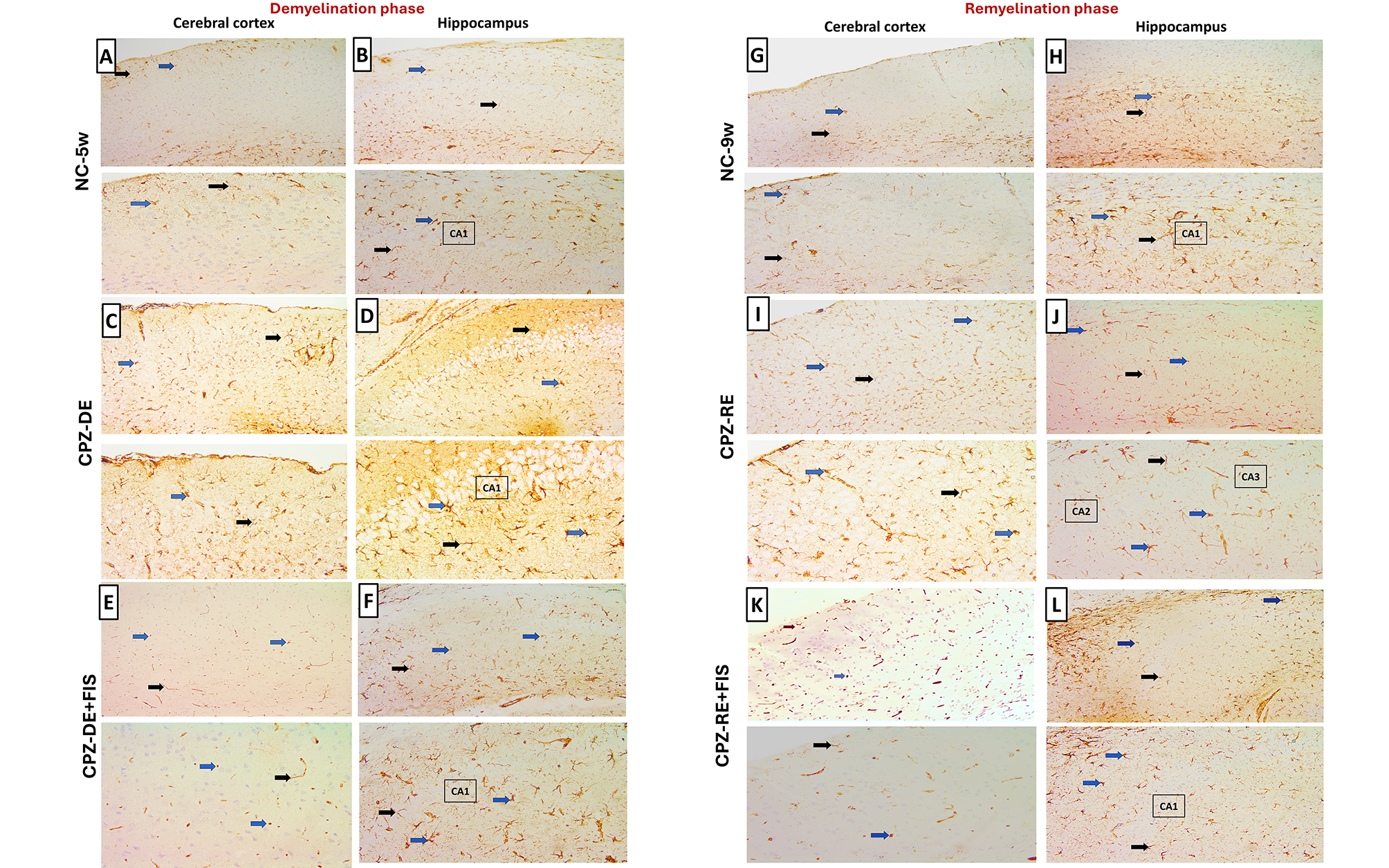

Supplement: Supplementary file 8 — (PNG 3.52 MB) [file 11481_2025_10260_Fig12_ESM.png]

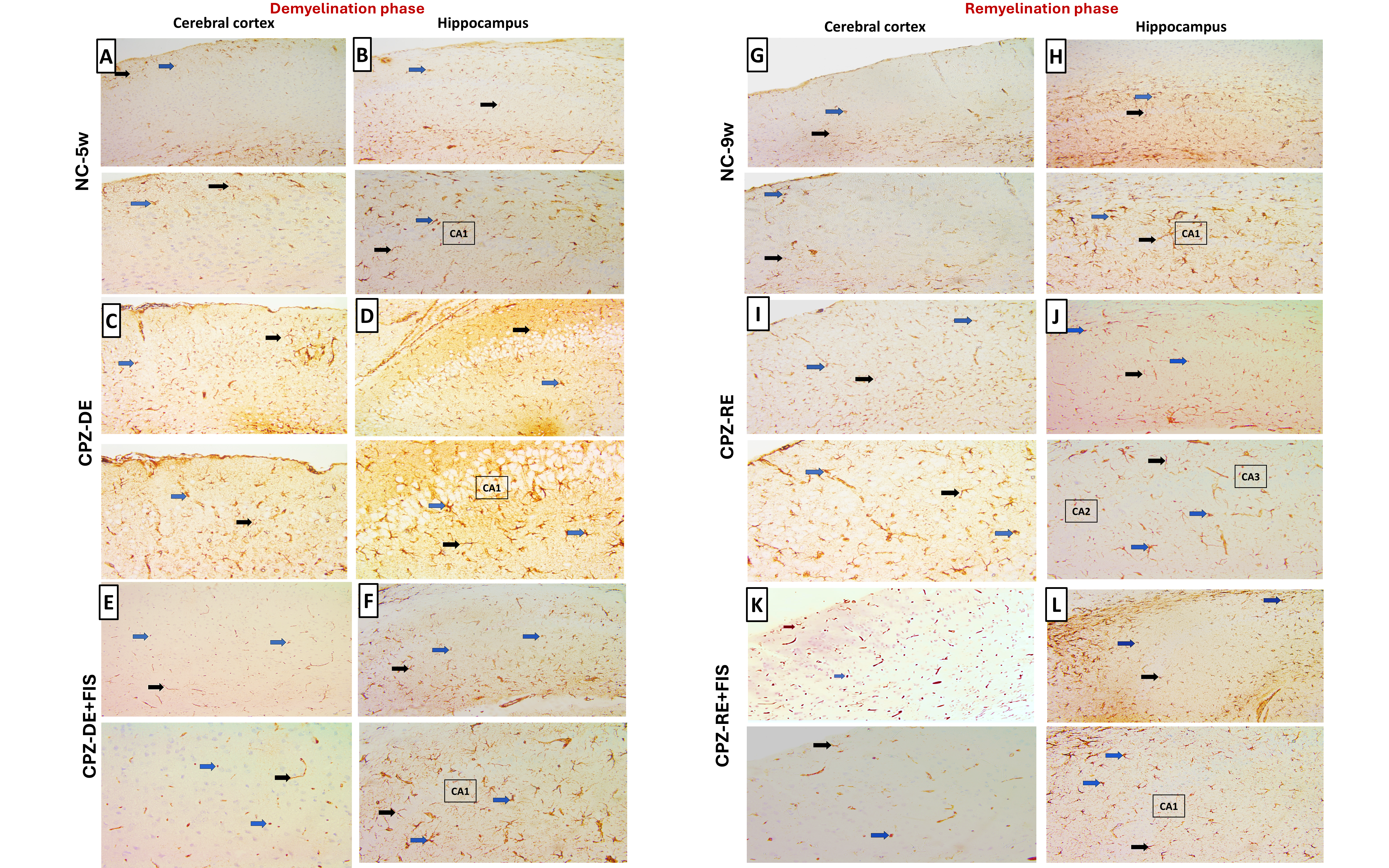

Supplement: Supplementary file 9 — High Resolution Image (TIF 42.5 MB) [file 11481_2025_10260_MOESM5_ESM.tif]

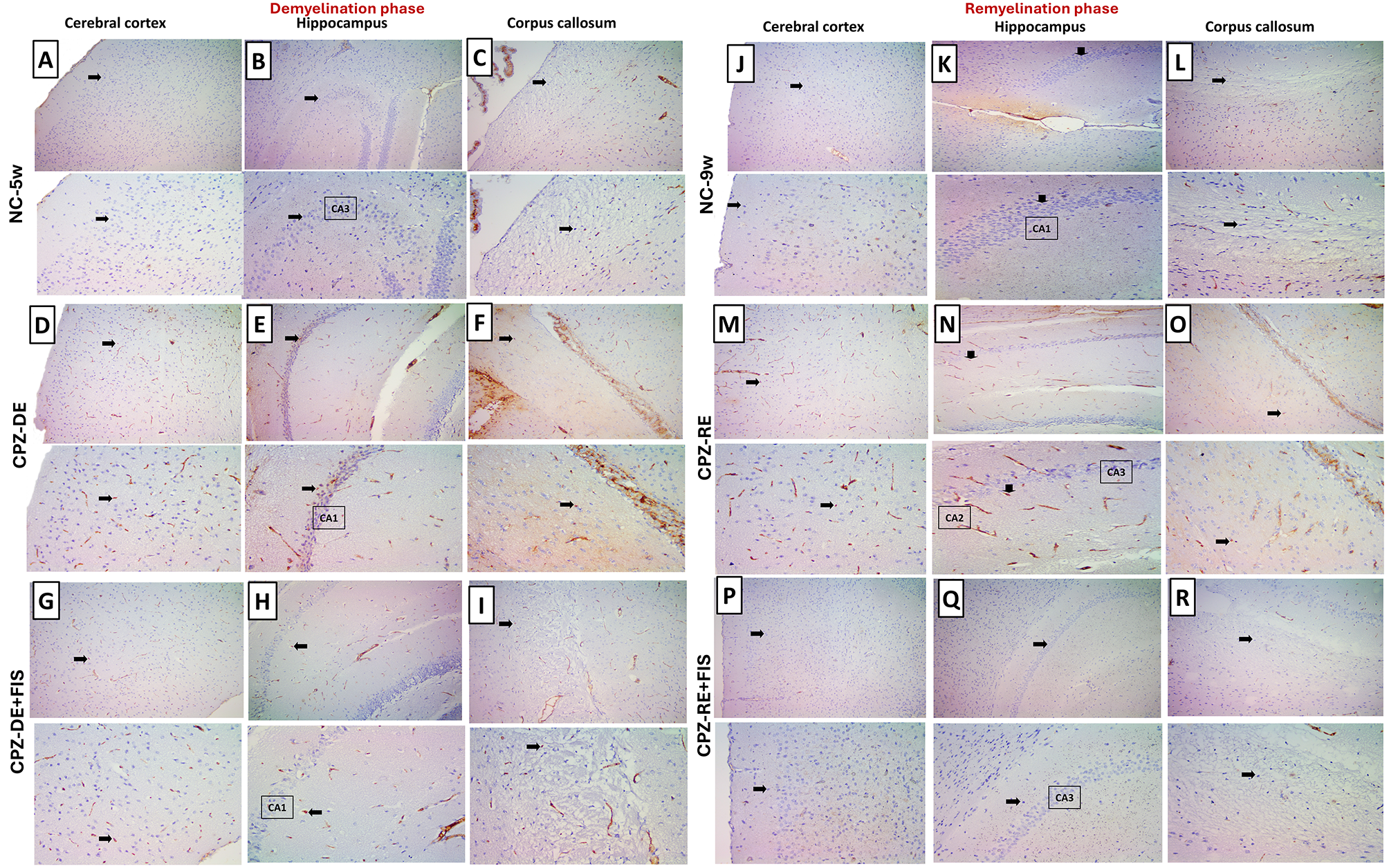

Supplement: Supplementary file 10 — (PNG 3.91 MB) [file 11481_2025_10260_Fig13_ESM.png]

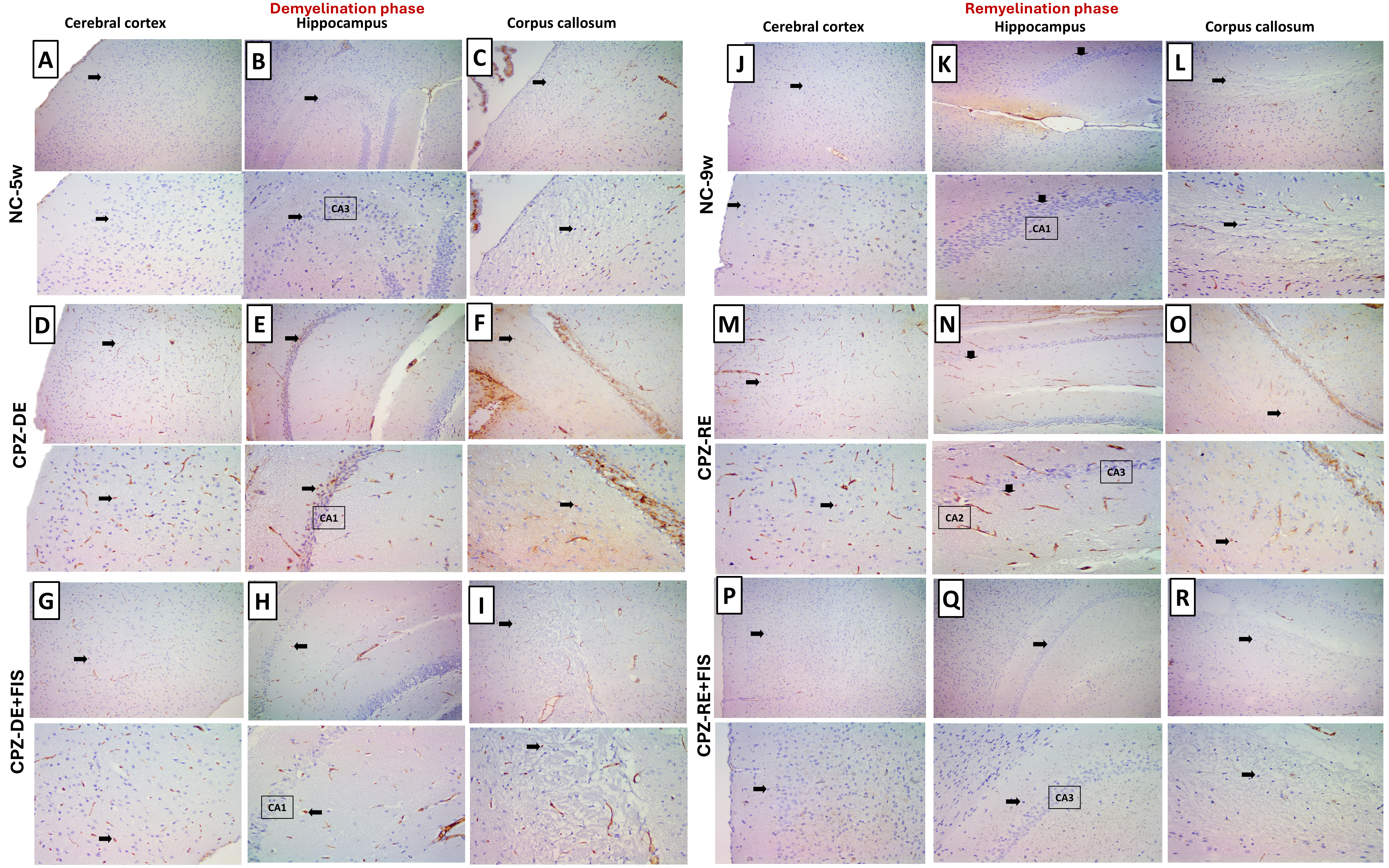

Supplement: Supplementary file 11 — High Resolution Image (TIF 43.6 MB) [file 11481_2025_10260_MOESM6_ESM.tif]
